# Synergistical Induction of Apoptosis via Cold Atmospheric Plasma and Nanohydroxyapatite for Selective Inhibition of Oral Squamous Cell Carcinoma in Tumour Microenvironment

**DOI:** 10.1111/cpr.70041

**Published:** 2025-04-29

**Authors:** Wenting Qi, Hanghang Liu, Huaze Liu, Yuxuan Guo, Li Wu, Chongyun Bao, Xian Liu

**Affiliations:** ^1^ State Key Laboratory of Oral Diseases & National Center for Stomatology & National Clinical Research Center for Oral Diseases West China Hospital of Stomatology, Sichuan University Chengdu Sichuan People's Republic of China; ^2^ Institute of Applied Electromagnetics College of Electronics and Information Engineering, Sichuan University Chengdu Sichuan People's Republic of China

**Keywords:** apoptosis, calcium, cold atmospheric plasma, nanohydroxyapatite, oral squamous cell carcinoma, reactive oxygen species

## Abstract

Surgical resection, radiotherapy and chemotherapy are the primary strategies of treating cancers globally. However, the current treatment methods bring new disease burdens to patients due to postoperative complications and multiple side effects, especially in surface tumours such as oral squamous cell carcinoma (OSCC). In this study, we developed a microwave cold atmospheric plasma (CAP) device in conjunction with tumour microenvironment‐responsive nanohydroxyapatite (nHA) for the first time. The synergistic effects of CAP and nHA combined application on OSCC were evaluated in both in vitro and in vivo experiments. The synergistic effects of CAP and pH‐responsive NH_2_‐nHA on the apoptosis, intracellular reactive oxygen species (ROS) and calcium ion concentration of OSCC cells were investigated in vitro. The synergistic induction of CAP with NH_2_‐nHA exhibited optimal tumour‐specific inhibitory effects on OSCC. The results revealed that the combined application of CAP with NH_2_‐nHA induced apoptosis of tumour cells in vitro and killed 84.0% of tumours in vivo. Mechanistically, CAP enhances extracellular ROS production, while NH_2_‐nHA amplifies intracellular calcium ion (Ca^2+^) concentrations, synergistically increasing intracellular ROS levels to provoke oxidative stress in OSCC cells, ultimately triggering the mitochondrial apoptosis pathway. In conclusion, the combined utilisation of CAP and NH_2_‐nHA presents a promising avenue as a novel, selective, and non‐invasive strategy in the management of OSCC.

## Graphical abstract

The synergistic effects of microwave‐induced CAP and pH‐responsive NH_2_‐nHA on OSCC cells were investigated, unveiling novel molecular mechanisms. The study highlights CAP+NH_2_‐nHA's dual action, inducing diverse apoptotic pathways and establishing a ROS‐Ca^2+^ amplifying loop, promising in cancer therapy.

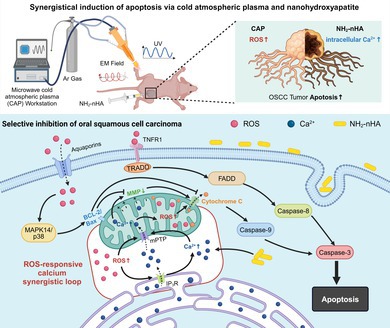

## 1 Introduction

Oral cavity cancer (OCC) is known to be the 16th most common malignancy in the world. The incidence and mortality rates of malignant tumours in the oral cavity have been steadily increasing globally, with 389,846 new cases and 188,438 deaths reported in 2021 (Global Cancer Observatory) [1]. Oral squamous cell carcinoma (OSCC) accounts for over 90% of these cases [2, 3]. Postoperative maxillofacial defects and side effects severely impact patients' physiological and psychological health, contributing to the highest risk of suicide among all cancer patients [4]. oSCC remains challenging to treat, requiring a multidisciplinary approach, with surgery, radiotherapy and systemic therapy serving as key components of the treatment of locally advanced disease [5, 6]. Despite these treatments, chemoresistance and immunosuppression hinder OSCC treatment, leading to unsatisfactory clinical outcomes for OSCC patients [7]. Additionally, existing treatments are hindered by complex equipment, stringent technical requirements and high costs, which impede timely diagnosis and treatment for some patients. Therefore, it is urgent to explore more selective methods of OSCC treatment.

Cold atmospheric plasma (CAP) has been successfully applied in more than 10 cancers, including lung carcinoma [8], breast cancer [9], melanoma [10, 11], colon cancer [12], cervical carcinoma [13], neuroblastoma [14], pancreatic carcinoma [15], and head and neck cancer (HNC) [16]. The therapeutic effect of CAP is related to high concentrations of reactive oxygen species (ROS) and reactive nitrogen species (RNS), including atomic nitrogen and oxygen, ozone (O_3_), hydroxyl (OH^−^), singlet delta oxygen (O_2_(a^1^Δ_g_)), superoxide (O_2_^−^), nitric oxide (NO), nitrogen dioxide (NO_2_) and nitrogen trioxide (NO_3_). CAP causes excess RONS to increase oxidative stress in cancer cells, inducing oxidative damage and resulting in cell death [17]. The mechanism underlying the anticancer effect of CAP is based on the metabolic characteristics of cancer cells. To date, several studies have shown that CAP has a selective effect on OSCC cell apoptosis both in vivo and in vitro [18]. Among these, there are two different ways of using CAP: direct exposure to the plasma jet and indirect exposure to the medium (plasma‐activated medium, PAM). Both methods decrease cancer cell viability and have demonstrated anticancer effects in different studies. Multiple studies have reported that intracellular ROS levels increase and that mitochondrial damage occurs after CAP treatment [19, 20, 21]. Some studies have reported cell cycle arrest in sub‐G1 or G2/M phases [21, 22, 23]. DNA damage is also observed [24]. CAP demonstrates great potential in cancer therapy by selectively inducing apoptosis in tumour cells. However, the synergy of CAP with other therapeutic modalities to achieve precise treatment of OSCC is not completely elucidated.

Hydroxyapatite [HA, Ca_10_(PO_4_)_6_(OH)_2_], a calcium‐based biomaterial, has been extensively used in orthopaedics and dentistry [25]. As the primary inorganic component of human bones and teeth, HA exhibits excellent bioactivity, osteoconductivity and biocompatibility. Additionally, it serves as a carrier for drug, protein and gene delivery [26, 27]. Numerous studies have demonstrated that nHA can inhibit the proliferation of various tumour cells and induce apoptosis, including osteosarcoma [28], malignant melanoma [29], breast cancer cells [30, 31, 32], gastric cancer cells [33, 34], colon cancer cells [35] and liver cancer cells [36]. Research indicates that nHA particles smaller than 100 nm exhibit superior efficacy in inhibiting tumour cell metabolic activity [29, 37]. Zhang et al. reported that a polyacrylic acid‐templated assembly of HAP nanoparticles was developed as an intracellular calcium generator for tumour‐specific therapy [34]. Research by Dong et al. further confirmed that the dissolution of nHA within tumour cells leads to intracellular calcium overload, thereby activating the mitochondrial apoptosis pathway and exerting tumour‐suppressive effects [38]. Thus, surface‐modified nHA may have the potential to target the tumour microenvironment to inhibit OSCC.

Research into new cancer treatments must be grounded in the physiological and metabolic characteristics of tumours to achieve selectivity and efficacy. The Warburg effect, a hallmark metabolic feature of tumours, manifests as increased glycolysis and reduced oxidative phosphorylation, leading to elevated lactate production and an acidic extracellular microenvironment [39]. Tumour cells exhibit high metabolic activity and elevated intracellular ROS levels. When subjected to exogenous stimuli, the ROS concentration in tumour cells can surpass their antioxidant capacity, triggering oxidative stress and apoptosis. In contrast, normal cells produce fewer ROS under physiological conditions and can mobilise reserve antioxidant enzymes to neutralise ROS upon exogenous stimulation, preventing their intracellular ROS levels from reaching apoptotic thresholds [40, 41]. Therefore, targeting ROS may provide a channel for the development of novel OSCC treatment strategies.

**SCHEME 1:**
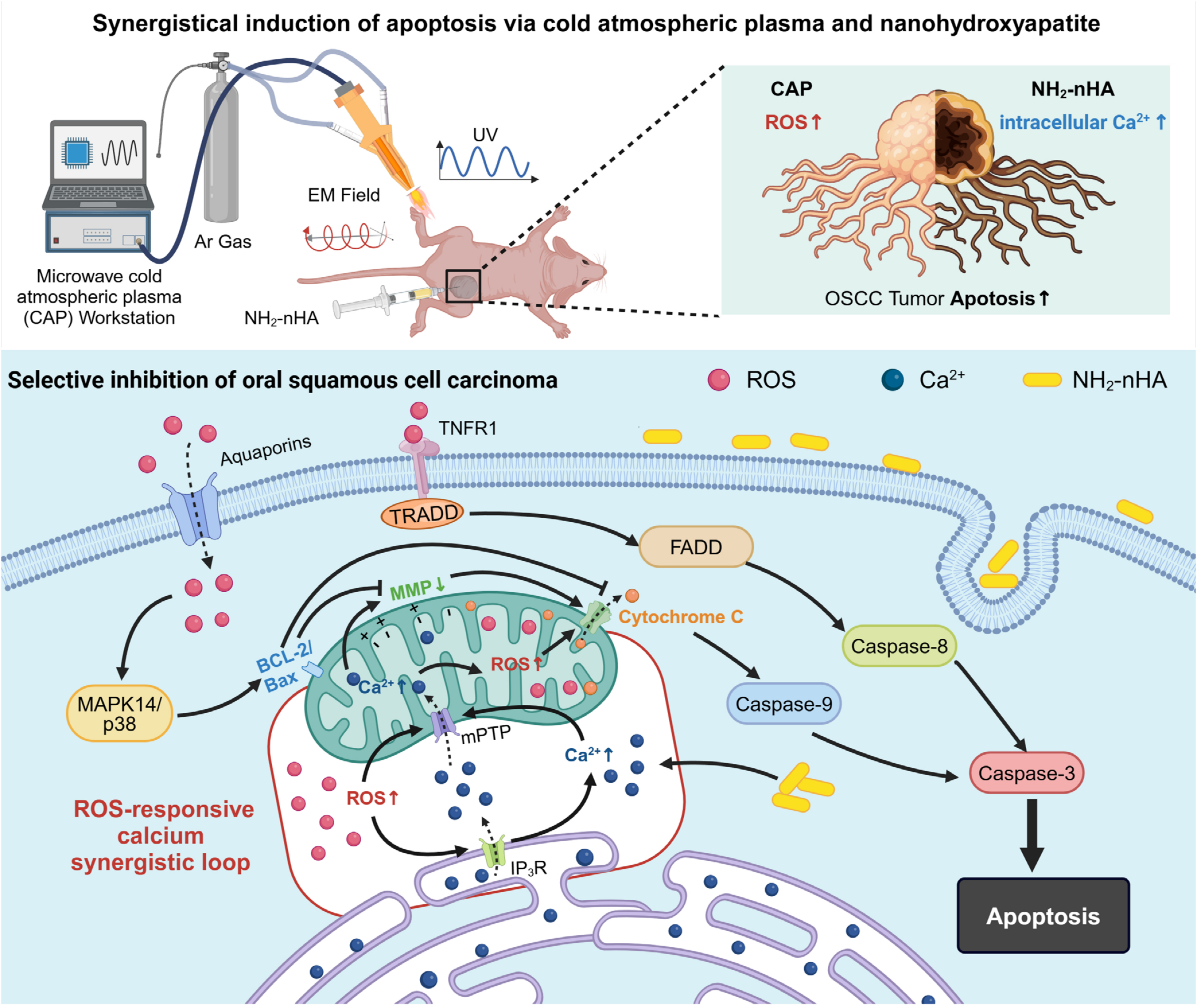
Illustration of the ROS‐responsive calcium synergistic loop for oral squamous cell carcinoma apoptosis induced by cold atmospheric plasma and NH_2_‐nHA synergistic intervention.

In this study, we utilise a portable microwave‐induced CAP device. The objective is to systematically compare the effects of CAP on the proliferation of HSC‐3 cells and L929 cells under different parameters and ways of action, to provide reference for the optimal mode and parameter of CAP for subsequent OSCC treatment. Moreover, CAP targeting ROS and pH‐responsive NH_2_‐nHA targeting calcium ions (Ca^2+^) were applied to HSC‐3 cells in vitro and in vivo to explore their effects on cell proliferation, apoptosis, cell cycle, mitochondrial function and transcriptional expression of related genes (Scheme 1). This study fills a critical gap in the investigation of CAP in conjunction with nanomaterials for tumour therapy, applicable in both in vitro and in vivo. Additionally, it is the first time to identify the mechanism of synergistic influence of CAP and NH_2_‐nHA on OSCC, aiming to provide potential reference for developing novel treatment strategies for OSCC.

## 2 Experimental

Shown in Supporting Information.

## 3 Results and Discussion

### 3.1 Direct CAP Showed Highly Selective Antiproliferative Effects in OSCC Cells

**FIGURE 1:**
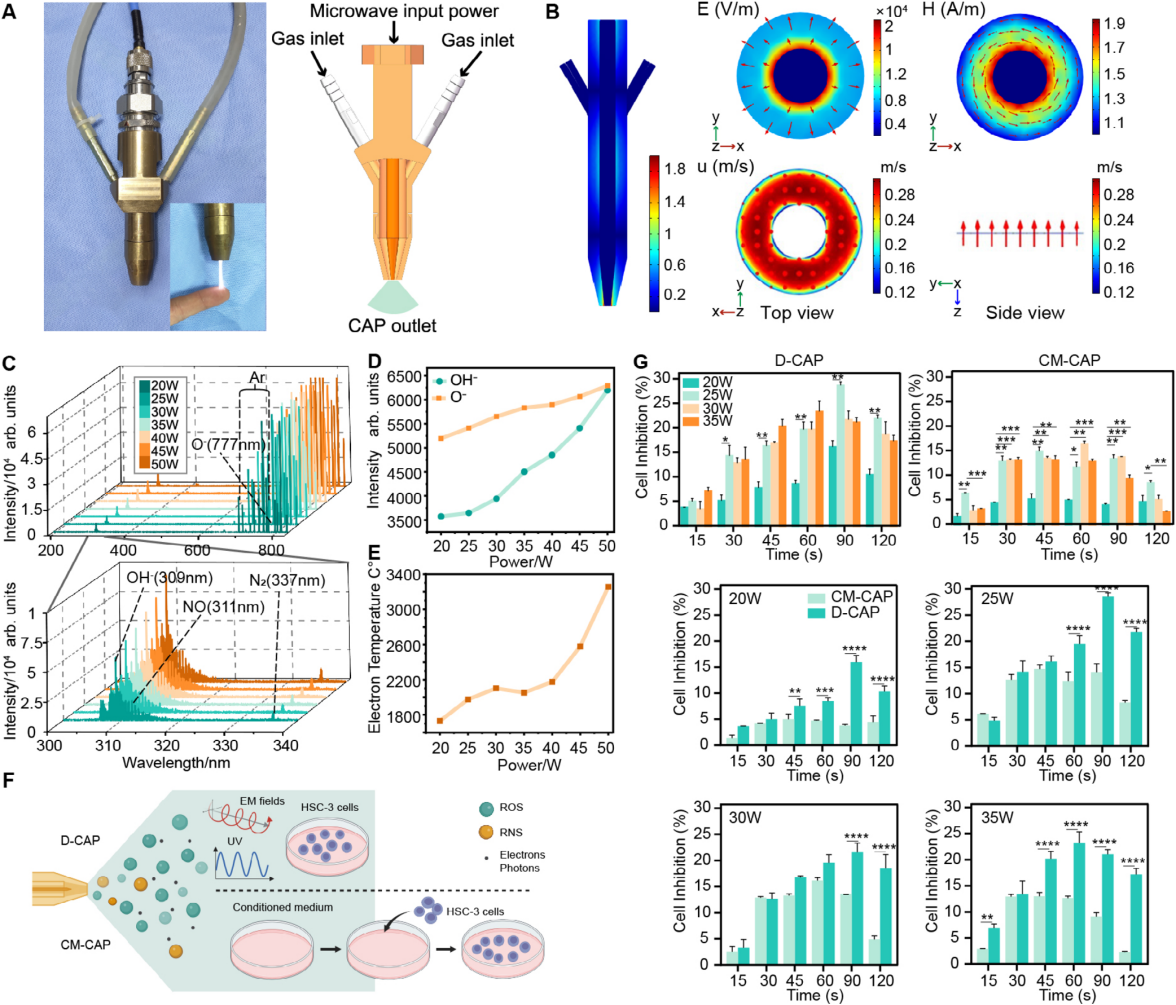
Structural diagram and component characterisation of CAP. (A) The structure of the plasma device and stable excitation of CAP. (B) The electromagnetic field distribution and the gas flow distribution at the nozzle. (C) The spectrum of the Ar CAP at different powers. (D) The intensity of OH^−^ and O^−^ at different powers. (E) The electron temperature at different powers. (F) Diagram of D‐CAP and CM‐CAP of CAP (created with BioRender.com). (G) Cell inhibition rate of HSC‐3 cells in the direct treatment group (D‐CAP) and culture medium treatment group (CM‐CAP) (not labelled: *p* > 0.05, *p ≤ 0.05, **p ≤ 0.01, ***p ≤ 0.001, ****p ≤ 0.0001).

A microwave CAP device was designed based on a coaxial structure with a gradually compressed open end, as shown in Figure 1A. Electromagnetic simulation and fluid simulation were uniformly distributed at the nozzle of this CAP device in Figure 1B. In Figure 1C, the emission spectrum analysis at different microwave powers (20–50 W) revealed that Ar was the dominant component (696–840 nm) [42]. ROS and RNS, key therapeutic agents, were detected, including OH^−^ radicals at 309 nm and atomic oxygen (O^−^) at 777 nm for ROS, and NO at 311 nm and nitrogen (N_2_) at 337 nm for RNS [43]. Figure 1D showed an increase in OH^−^ intensity with rising power, indicating heightened microwave input boosts OH^−^ and O^−^ concentrations. OH^−^ was noted as the most potent oxidizer among these ROS, suggesting higher power plasma jets may enhance treatment efficacy [44].

To determine the optimal application of CAP, we examined its effects on HSC‐3 cells in two ways: direct CAP (D‐CAP) and CAP‐activated culture medium (CM‐CAP), as depicted in Figure 1F. The D‐CAP group was exposed to a blend of ROS, RNS, electromagnetic (EM) fields, and UV, while the CM‐CAP group received only ROS and RNS. Results in Figure 1G showed both D‐CAP and CM‐CAP inhibited HSC‐3 cell proliferation, while D‐CAP exhibited significantly greater inhibition (3.28%–26.94%) compared to CM‐CAP (2.47%–16.18%). Interestingly, D‐CAP 25 W 90 s exhibited the best OSCC inhibitory effect. Hence, D‐CAP emerges as a superior approach for OSCC treatment compared to CM‐CAP. Therefore, D‐CAP at 25 W for 90 s was selected as the optimal setting for CAP treatment in this study. To investigate the effective duration of CAP treatment, the cell inhibition rate was analysed from 1 to 5 days post‐treatment. In Figure S1, the highest HSC‐3 cell inhibition rate of 35.63% was observed on the third day for the 90 s group. These results indicate that the 25 W 90 s CAP treatment exhibited the most effective OSCC cell inhibition on Day 3. The inhibitory effect of CAP is not time‐ and dose‐dependent. In breast cancer studies by other researchers, that 30–90 s CAP treatments lead to more significant reductions in viability than 180 s CAP exposure [45].

As the electron temperature raised with increasing power levels of CAP in Figure 1E, does the remarkable effectiveness of D‐CAP treatment on OSCC cells correlate with temperature elevation? To address this question, thermal imaging was employed to monitor temperature changes in the 6‐well plate following CAP exposure ranging from 15 to 180 s with 35 W in Figure S2. High‐temperature electrons colliding with the plastic well walls contributed to heating the plate, causing adjacent control group temperatures to rise. After 180 s of CAP treatment, the highest temperatures recorded were 32.55°C on the plastic well walls. Due to Ar gas airflow dissipating heat, the CAP group's culture medium temperature gradually decreased from 29.12°C to 23.98°C between 15 and 90 s. In summary, the thermogenesis of D‐CAP could not cause the temperature rise of the medium to induce thermal damage to inhibit OSCC cells. To further determine whether CAP has a selective inhibitory effect on tumour cells, L929 cells (a mouse fibroblast line) were treated with D‐CAP. The results showed that D‐CAP under all parameters promoted the viability of L929 cells (Figure S3). These findings align with previous studies indicating CAP treatment's promotion of collagen deposition and epithelial proliferation, thereby facilitating wound healing [46, 47]. Thus, CAP acts as a selective therapy with a high affinity for inhibiting OSCC cells while leaving normal cells unharmed.

### 3.2 CAP Induced Apoptosis and Cell Cycle Arrest

**FIGURE 2:**
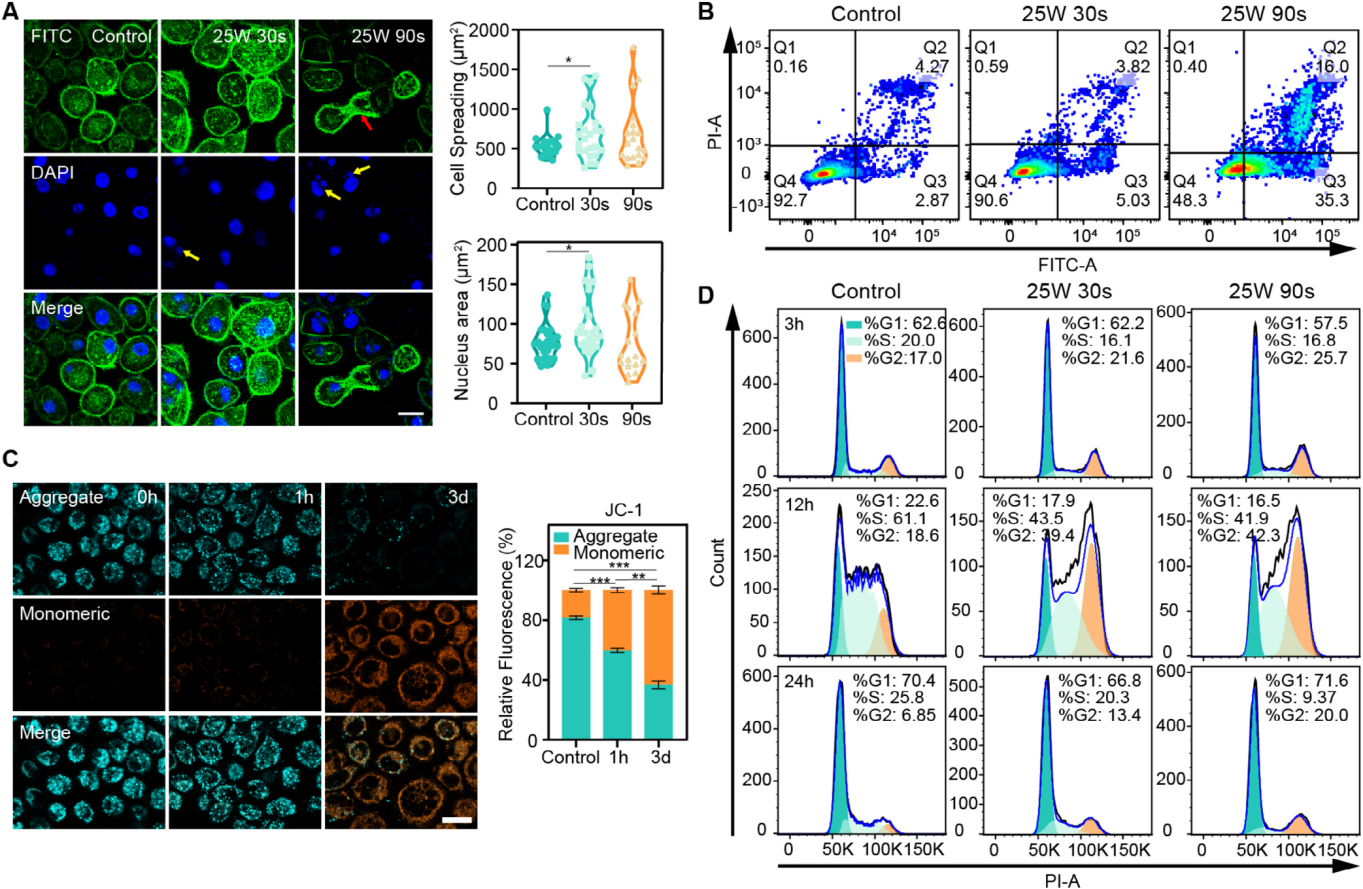
Cell morphology, cell cycle progression and apoptosis after CAP treatment of HSC‐3 cells. (A) Fluorescence images of cytoskeleton staining of HSC‐3 cells after CAP treatment on 3 days. Cytoskeleton: F‐actin (green). Nuclei: DAPI (blue) (scale bar 20 μm). Average cell spreading area and nucleus area normalised per cell. (B) Flow cytometry analysis of the apoptosis of HSC‐3 cells induced by CAP treatment at different powers for 24 h (Statistical analysis results were shown in Figure S4). Q1, necrotic cells; Q2, late apoptotic cells; Q3, early apoptotic cells; Q4, living cells. (C) Fluorescence images of change in mitochondrial transmembrane potential (ΔΨm) in HSC‐3cells after CAP treatment 25w 90s: JC‐1 aggregates (cyan), JC‐1 monomers (orange). JC‐1 aggregates are more in control cells revealing intact mitochondria and the formation of JC‐1 monomers in CAP treated cells shows dissipation of ΔΨm. Increase in JC‐1 monomers and decrease in JC‐1 aggregates in CAP‐treated cells shows the capability to damage mitochondria (scale bar 20 μm). Quantification of relative intensity of JC‐1 monomers and JC‐1 aggregates fluorescence. (D) The effect of CAP treatment on cell cycle progression in HSC‐3 cells at different powers for 3 h to 24 h (Statistical analysis results were shown in Figure S5) (not labelled: *p* > 0.05, *p ≤ 0.05, **p ≤ 0.01, ***p ≤ 0.001).

Typical hallmarks of apoptosis included cell shrinkage, convolution of the nuclear and cellular outlines, cell membrane blebbing, the presence of apoptotic bodies and chromatin condensation [48]. In contrast to apoptosis, necrosis is characterised by swelling, early plasma membrane rupture, and disruption of cellular organelles, including mitochondria. In addition, the formation of apoptotic bodies and budding is absent [49]. As the changes in cell morphology and nucleus shown in Figure 2A, after CAP treatment, the cell membrane remained continuous and intact, while the cell morphology exhibited irregularities, convolution, and karyorrhexis. In the 90 s group, a large number of cells demonstrated karyorrhexis (yellow arrow), and apoptotic bodies (red arrow) containing plasma were observed. Based on previous reports, the morphological characteristics observed suggest that apoptosis may be induced in HSC‐3 cells under CAP treatment.

After CAP treatment, Annexin V‐FITC and propidium iodide (PI) flow cytometric staining analysed cell death. In the CAP group, early and late apoptotic cell numbers increased; the 90s group had a significantly higher early apoptotic cell percentage than the control (35.3% vs. 2.87%). The apoptotic ratio rose dose‐dependently with exposure time. Inhibiting cancer cells often involves interfering with the cell cycle [50, 51]. Since cancer cells proliferate faster and have more S phase cells than normal cells, studies suggest CAP modifies the cell cycle [40]. To clarify CAP's antiproliferative mechanism, we studied its effect on HSC‐3 cell cycle progression. HSC‐3 cells treated with CAP were stained with PI for DNA and analysed by flow cytometry at 3, 12, and 24 h in Figure 2D. CAP treatment for 30 and 90 s tripled G2/M phase cell numbers compared to the control, indicating G2/M phase arrest. Consistent with the results, Blackert et al. showed that CAP could induce increased amounts of ROS produced by plasma that might cause oxidative DNA damage in keratinocytes and delay the cell cycle (G2/M phase arrest), corresponding to a loss of viability [52]. Volotskova et al. also presented that CAP induced a robust G2/M increase in two different types of cancer cells with different degrees of tumorigenicity [40].

Maintaining mitochondrial membrane potential (MMP) is vital for mitochondrial function, and depolarization occurs early in apoptosis. To determine when CAP induced apoptosis starts, we measured MMP at 0, 1, and 3 days post CAP treatment. In Figure 2C, MMP decreased as early as 1 h post CAP treatment, with monomers increasing from 18.6% to 42.2%, and significantly more on the 3 days (64.7%). These results show CAP promptly induces mitochondrial dysfunction, early apoptosis, and G2/M phase arrest in HSC‐3 cells.

### 3.3 CAP Induced an Increase in Intracellular Ca^2+^ and ROS to Activate Apoptosis Pathway

**FIGURE 3:**
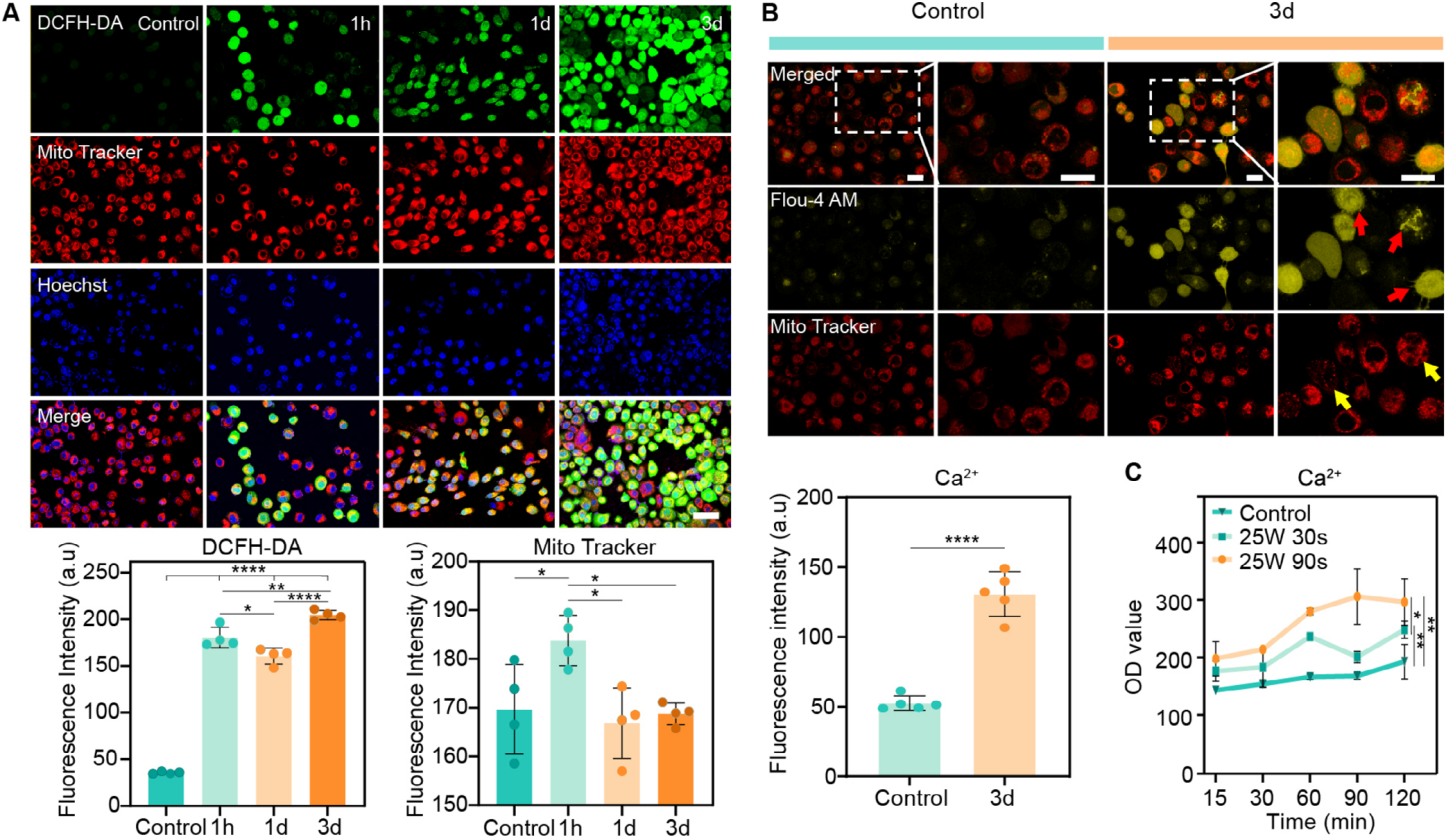
Fluorescence image of intracellular ROS, intracellular Ca^2+^, mitochondria after CAP treatment of HSC‐3 cells. (A) CLSM image of HSC‐3 cells after CAP treatment for 1 h to 3 days: Alteration of the intracellular ROS levels after CAP treatment (DCFH‐DA: green); fluorescence intensity of bioactive mitochondria (Mito‐Tracker Red CMXRos: red); nucleus stained with Hoechst (blue) (scale bar 50 μm). Quantification of the intensity of DCFH‐DA surface fluorescence. Quantification of MitoTracker surface fluorescence intensity. (B) The colocalization of intracellular Ca^2+^ with mitochondria in HSC‐3 cells after CAP treatment for 3 days (scale bar 20 μm). Quantification of the intensity of the Fluo‐4 AM fluorescence. (C) The OD value was used to analyse the intracellular calcium concentration of cells exposed to CAP for 15 min to 2 h. The results are expressed as the OD value ± SD (not labelled: *p* > 0.05, *p ≤ 0.05, **p ≤ 0.01, ****p ≤ 0.0001).

Hyperproliferation of tumour cells produces excessive ROS, leading to the aberrant redox status of tumour cells. But excessive ROS levels are cytotoxic. To determine the effects of CAP on intracellular ROS levels and mitochondria, HSC‐3 cells were treated with CAP for 90 s and stained with DCFH‐DA and Mito Tracker (MitoTracker Red CMXRos). As shown in Figures 3A and S6, at 1 and 3 days, a large number of cells with high intracellular ROS levels were observed, and the ROS levels peaked at 3 days. After CAP treatment for 1 h, an increase in the intracellular ROS concentration was observed in a few cells. The fluorescence intensity of the mitochondria was greatest at 1 h, suggesting that functional aggregation of the mitochondria might have occurred at the same time. To thrive under oxidative stress, tumour cells increase their antioxidant status to optimise ROS‐driven proliferation, while breaking the ROS thresholds that would trigger senescence or apoptosis [53]. Combined with the increased loss of mitochondrial membrane potential observed at 1 h in Figure 2C, it could be inferred that CAP treatment facilitates ROS penetration into the cells, inducing mitochondrial depolarisation and subsequent apoptosis.

Calcium (Ca^2+^) is an important second messenger involved in intra‐ and extracellular signalling cascades and plays an essential role in cell life and death decisions [54]. Calcium signalling and ROS signalling are closely related. As shown in Figure S7, after CAP treatment, the Fluo‐4 AM fluorescent probe was used to detect intracellular calcium ions in HSC‐3 cells at 1 and 2 h. Compared with that in the control group, an increase in the intracellular Ca^2+^ concentration was observed 15 min after CAP treatment, and the intracellular Ca^2+^ concentration in the 90 s group reached its peak after 90 min in Figure 3C. Compared with that in the 30 s group, the intracellular Ca^2+^ concentration in the 90 s group was greater. Figure 3B shows the colocalisation of intracellular Ca^2+^ with mitochondria, indicating that the intracellular Ca^2+^ concentration in HSC‐3 cells after CAP treatment for 3 days was significantly greater than that in the control group. Compared with that in the control group, the intracellular Ca^2+^ in the cytoplasm was more evenly distributed. Mitochondria play a pivotal role in the control of apoptosis and are involved not only in the intrinsic pathway but also in the extrinsic pathway. In cells with high Ca^2+^ concentrations, mitochondria form aggregates and dense masses, which are features of apoptosis. Previous research has shown that mitochondria are uniformly distributed throughout the cytoplasm and exhibit a typical elongated shape in control cells. In apoptotic cells, mitochondria become fragmented, forming aggregates and more densely packed masses in the cytoplasm [55].

Previous research has indicated that excessive accumulation of ROS can lead to the oxidation of ER‐resident Ca^2+^ release channels, such as ryanodine receptors (RyRs) in excitable cells and IP3‐R in no excitable cells. Oxidation enhanced channel activity and increased Ca^2+^ release from the ER [56]. Ca^2+^ overload resulted in transient opening of the mitochondrial permeability transition pore (mPTP), and the accumulation of ROS leads to sustained mPTP opening [57]. According to pioneering researcher Kroemer et al., the permeabilisation of mitochondrial membranes is a decisive step in apoptosis [58]. Then, Ca^2+^ is transported to the mitochondria through the mPTP in the MAM. Increased Ca^2+^ influx into mitochondria activates ROS generation and results in the depolarization of the MMP, with subsequent release of cytochrome C and activation of several calcium‐dependent protein kinases [59, 60]. Taken together, CAP treatment induces apoptosis via an increase in intracellular ROS and mitochondrial dysfunction. Remarkably, ROS‐mediated calcium overload and calcium‐induced ROS increases create a ROS‐Ca^2+^ synergistic amplifying loop, leading to the prolonged activation of mitochondria‐dependent apoptosis.

### 3.4 Tumour Microenvironment‐Responsive NH_2_ ‐nHA Exerted Antiproliferative Effects in OSCC Cells

**FIGURE 4:**
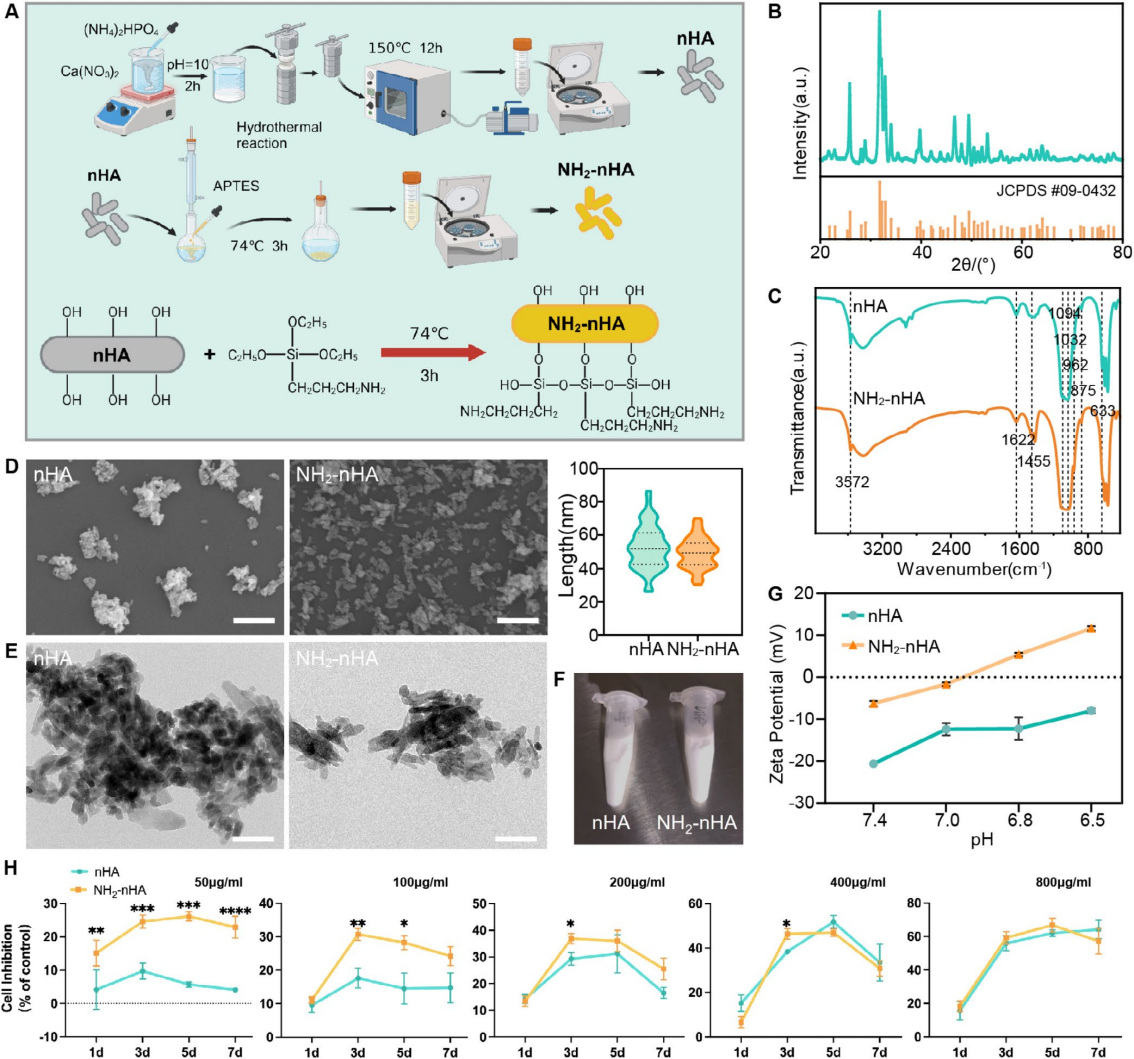
Preparation and characterisation of the materials. (A) Schematic diagram of the material preparation process. (B) X‐ray diffraction pattern of nHA. (C) FTIR spectra of nHA and NH_2_‐nHA. (D) SEM image and particle size statistical results of nHA and NH_2_‐nHA (scale bar 2 μm, 400 nm). (E) TEM image of nHA and NH_2_‐nHA (scale bar 100 nm). (F) Pictures of nHA and NH_2_‐nHA. (G) Zeta potential of nHA and NH_2_‐nHA at different pH. (H) The effect of different concentrations of nHA and NH_2_‐nHA on the viability of HSC‐3 cells (not labelled: *p* > 0.05, *p ≤ 0.05, **p ≤ 0.01, ***p ≤ 0.001, ****p ≤ 0.0001).

The preparation process of nHA and NH_2_‐nHA was briefly described in Figure 4A. The physicochemical properties and morphology of the nanoparticles were characterised by XRD, FTIR, SEM, TEM and zeta‐potential. The crystal phase structure of nHA detected by XRD was shown in Figure 4B, and the diffraction peak was similar to that of pure HA (Joint Committee on Powder Diffraction Standards, JCPDS, #09‐0432). Then the chemical structure of nHA and NH_2_‐nHA nanoparticles was analysed with FTIR to characterise the functional groups. As shown in Figure 4C, the peaks around 3572 and 633 cm^−1^ were the vibration absorption of the O—H bond of the hydroxyl group in the samples. The peaks around 1094, 1032, 962, 603 and 565 cm^−1^ were caused by P—O stretching vibration and bending vibration in PO_4_^3−^ and HPO_4_^3−^. The —NH— was observed at 1455 cm^−1^ in the NH_2_‐nHA sample. The SEM and TEM images of nHA and NH_2_‐nHA were mostly short rod‐like in Figure 4D,E. The nHA and NH_2_‐nHA were both white nanoparticles, with particle sizes at a distribution of 30–80 nm (Figure 4D,F).

Tumours display the Warburg effect, favouring glycolysis over oxidative phosphorylation, leading to increased lactate production. Lactate anions make tumour cells negatively charged, and the tumour microenvironment has a pH of 6.5–6.8 [39, 61]. The surface potentials of both nHA and NH_2_‐nHA in Figure 4G were negative at pH 7.4 and 7.0, while the surface potentials of NH_2_‐nHA turned positive under the tumour microenvironment. Therefore, NH_2_‐nHA shows surface potential changes in response to the tumour microenvironment.

The inhibition of nHA and NH_2_‐nHA nanoparticles was evaluated by CCK 8 assay in HSC‐3 cells with different concentrations. As shown in Figure 4H, nHA and NH_2_‐nHA nanoparticles had a concentration‐dependent effect on inhibition. When the concentration exceeded 200 μg/mL, there was no statistically significant difference in the inhibition rate between the two groups. While the nanoparticles concentration was at 50 μg/mL, the difference in OSCC inhibition rate between nHA and NH_2_‐nHA was the most significant. Therefore, a concentration of 50 μg/mL was used as the experimental concentration in the subsequent experiments. The results in Figure S8 indicated that nHA and NH_2_‐nHA could enter and be retained within OSCC cells. These results demonstrated that nHA and NH_2_‐nHA were phagocytosed by HSC‐3 cells to exert inhibitory effects. In particular, NH_2_‐nHA showed a more efficient and sustained inhibitory effect on HSC‐3 at low concentrations, highlighting the effectiveness of NH_2_‐nHA in tumour microenvironment response.

The surface potential of nano‐hydroxyapatite (nHA) influences cell adhesion and cellular behaviour. Palcevskis et al. found that negatively charged nHA has enhanced adhesion to osteoblasts [62]. Dekhtyar et al. showed that a 0.2 V increase in nHA surface potential improved osteoblast adhesion, boosting bone tissue formation, especially when nHA was negatively charged [63]. Here, NH_2_‐nHA particles carried a negative charge in body fluid environments, but it switched to a positive charge in the acidic tumour microenvironment. This change allowed NH_2_‐nHA to effectively adhere to the negatively charged tumour cell surfaces, facilitating phagocytosis and subsequently exhibiting inhibitory effects to OSCC cells.

### 3.5 NH_2_ ‐nHA and CAP Synergistically Increased Ca^2+^ Concentration and Induced Apoptosis of OSCC Cells

**FIGURE 5:**
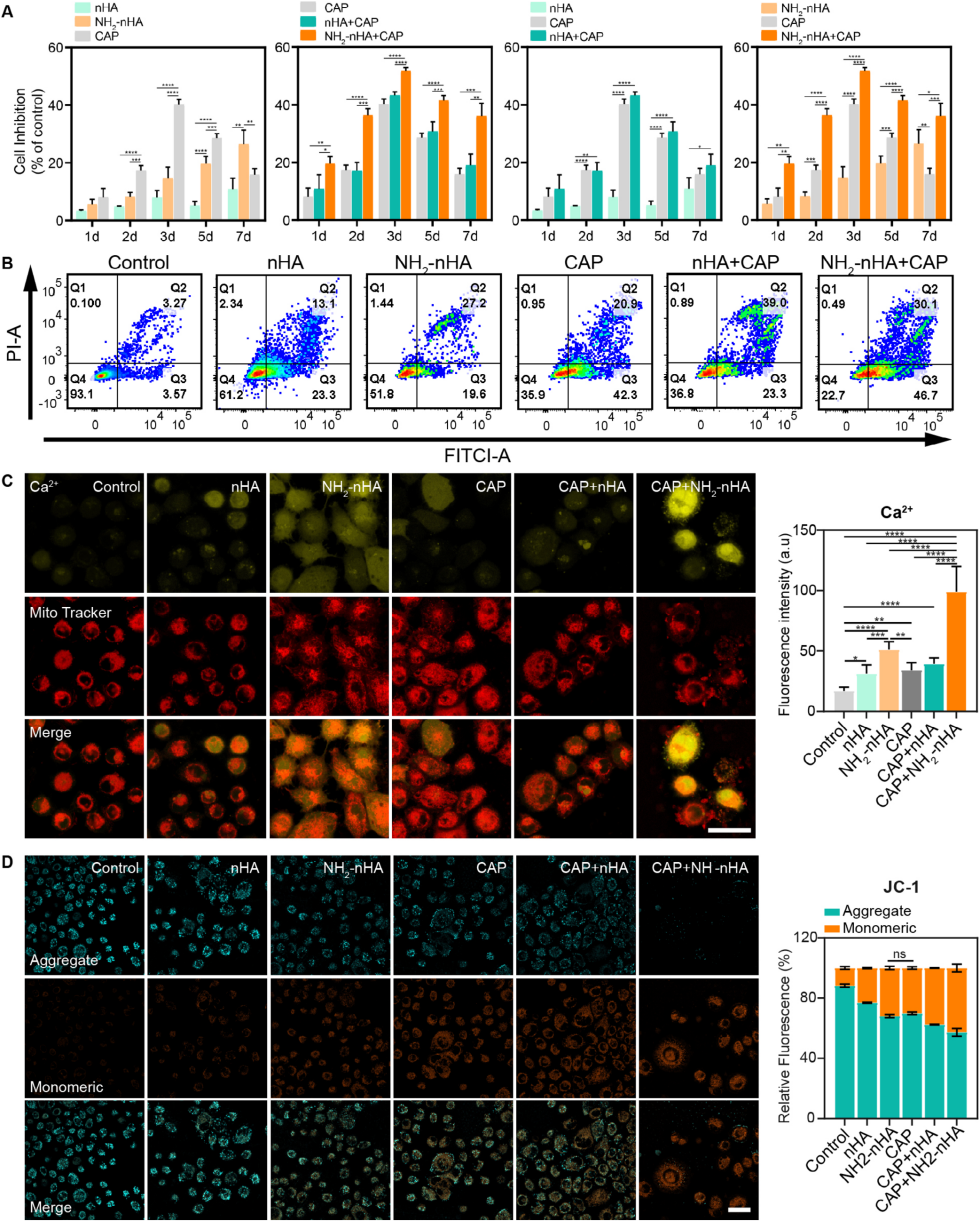
Cell viability, apoptosis and cell morphology after CAP, nHA and NH_2_‐nHA synergistically treatment of HSC‐3 cells. (A) The effect of synergistic intervention with nHA, NH_2_‐nHA and CAP on the viability of HSC‐3 cells (not labelled: *p* > 0.05, *p ≤ 0.05, **p ≤ 0.01, ***p ≤ 0.001, ****p ≤ 0.0001). (B) Flow cytometry analysis of the apoptosis of HSC‐3 cells induced by synergistic intervention with nHA, NH_2_‐nHA and CAP on 3 days (Statistical analysis results were shown in Figure S11). Q1, necrotic cells; Q2, late apoptotic cells; Q3, early apoptotic cells; Q4, living cells. (C) Fluorescence images of intracellular Ca^2+^ and mitochondria in HSC‐3 cells after 24 h of treatment (scale bar 40 μm). Statistical analysis of intracellular Ca^2+^ fluorescence intensity in each group (not labelled: *p* > 0.05, *p ≤ 0.05, **p ≤ 0.01, ***p ≤ 0.001, ****p ≤ 0.0001). (D) Fluorescence images of changes in mitochondrial membrane potential (ΔΨm) in HSC‐3 cells for 24 h: JC‐1 aggregates (cyan), JC‐1 monomers (orange) (scale bar 40 μm). Statistical results of relative fluorescence intensity of JC‐1 monomers and aggregates (ns indicates no significant statistical difference, not labelled: ****p ≤ 0.0001).

To investigate the synergistic anticancer effect of CAP with nHA or NH_2_‐nHA, Figure 5A shows that from 1 to 5 days, CAP had a higher cell‐inhibition rate than nHA or NH_2_‐nHA, peaking at 40.2% on Day 3, indicating its potent yet short‐lived effect on HSC‐3 cells. NH_2_‐nHA's inhibitory effect on HSC‐3 cells was consistently greater than nHA's, increasing from 1 to 7 days to 26.46% (exceeding CAP's 15.92% on Day 7), suggesting a persistent inhibitory effect. Combining nHA or NH_2_‐nHA with CAP increased the inhibition rate. For example, on Day 3, NH_2_‐nHA's rate was 14.7%, rising to 51.68% with CAP. Figure S9 shows no significant difference in inhibition rate due to different intervention sequences of CAP and nHA or NH_2_‐nHA. These suggest CAP and NH_2_‐nHA may complement each other over time, with CAP having a strong but brief effect and NH_2_‐nHA a prolonged one. Thus, the combination of nHA or NH_2_‐nHA with CAP significantly boosts the inhibitory effect on OSCC cells, and the NH_2_‐nHA+CAP synergistic intervention exerted the optimal tumour inhibition effect.

Then, apoptosis detection was performed at 3 days after intervention in each group in Figure 5B. It turned out that NH_2_‐nHA+CAP had the most remarkable induction of apoptosis in HSC‐3 cells, up to 76.8%. Compared to the nHA group 36.4%, the NH_2_‐nHA group had a proportion of apoptotic cells of 46.8%. Meanwhile, HSC‐3 cell skeleton staining was performed to observe cell morphology and structure in Figure S10.

Considering the above results and conclusions, the mechanism of NH_2_‐nHA and CAP synergistically promoting apoptosis of OSCC is still unclear. nHA and NH_2_‐nHA were phagocytosed by cells and degraded into Ca and P in lysosomes under acidic conditions. Therefore, we used fluorescence probes to stain intracellular Ca^2+^ concentration and mitochondria in Figure 5C. The CAP enhanced intracellular Ca^2+^ concentration in part of the cells, while significant Ca^2+^ concentration enhancement was observed in most cells in both NH_2_‐nHA and NH_2_‐nHA+CAP groups. Thus, we concluded that intracellular Ca^2+^ levels in all experimental groups were higher than in the control group, indicating the impact of interventions on HSC‐3 cells. The NH_2_‐nHA group had a significantly higher intracellular Ca^2+^ concentration than the nHA group, further indicating that NH_2_‐nHA was more likely to enter the HSC‐3 cells and released more Ca^2+^. This led to the conclusion that NH_2_‐nHA affected mitochondrial function by increasing intracellular Ca^2+^ levels, ultimately leading to apoptosis in HSC‐3 cells. The CAP+NH_2_‐nHA group had much higher intracellular Ca^2+^ levels than the CAP and CAP+nHA groups, suggesting that the combination of CAP and NH_2_‐nHA exerts a better synergistic effect by increasing intracellular Ca^2+^ levels, providing a basis for activating apoptosis.

Mitochondria are the powerhouses of all cells, so their function is closely tied to cellular status. Changes in mitochondrial membrane potential are an early hallmark of apoptosis [64]. In the nHA, NH_2_‐nHA, CAP, nHA+CAP, and NH_2_‐nHA+CAP groups, the mitochondrial membrane potential decreased and depolarised after 24 h of intervention in Figure 5D. The fluorescence images indicated that nHA and NH_2_‐nHA also induced apoptosis by affecting mitochondrial function, similar to CAP‐induced apoptosis in HSC‐3 cells. The CAP+NH_2_‐nHA group exhibited the highest proportion of mitochondrial membrane potential loss, indicating that it has the optimal capability to induce apoptosis.

It was hypothesized that the reason for the above result is that functionalized NH_2_‐nHA alters the surface potential of nHA, increasing its adhesion to cells, making NH_2_‐nHA more adherent to HSC‐3 cells than nHA. Subsequently, CLSM was used to observe the fluorescence images of nHA, NH_2_‐nHA and mitochondria (Mito Tracker), as shown in Figure S8. The nHA and NH_2_‐nHA were observed in HSC‐3 cells, confirming that nHA and NH_2_‐nHA could be phagocytosed by HSC‐3 cells. Remarkably, the NH_2_‐nHA adhered around the cells, confirming the response of NH_2_‐nHA to the tumour microenvironment. In addition to the control group, mitochondria became fragmented, forming aggregates and more densely packed masses in the cytoplasm, indicating early apoptosis in the cells.

Thus, functionalized NH_2_‐nHA promotes apoptosis of HSC‐3 cells significantly and speculates that the surface potential change of NH_2_‐nHA effectively increased cell adhesion and nanoparticles phagocytosis. CAP combined with NH_2_‐nHA promoted apoptosis synergetically in the most notable way. The NH_2_‐nHA, nHA+CAP, and NH_2_‐nHA+CAP groups showed elongated cell morphology and mitochondrial membrane potential loss, indicating early apoptosis. Early apoptotic cells were observed in both nHA and NH_2_‐nHA groups, confirming that HSC‐3 cells phagocytose nHA and NH_2_‐nHA. Additionally, the NH_2_‐nHA and NH_2_‐nHA+CAP groups show granular green fluorescence surrounding the cells, confirming that NH_2_‐nHA has a higher adsorption capacity to HSC‐3 cell surfaces than nHA. Therefore, NH_2_‐nHA is more effective than nHA in inducing apoptosis in HSC‐3 cells, and NH_2_‐nHA+CAP is more effective than nHA+CAP.

### 3.6 Functional Enrichment Analysis and Western Blot of NH_2_ ‐nHA and CAP Treatment HSC‐3 Cells

**FIGURE 6:**
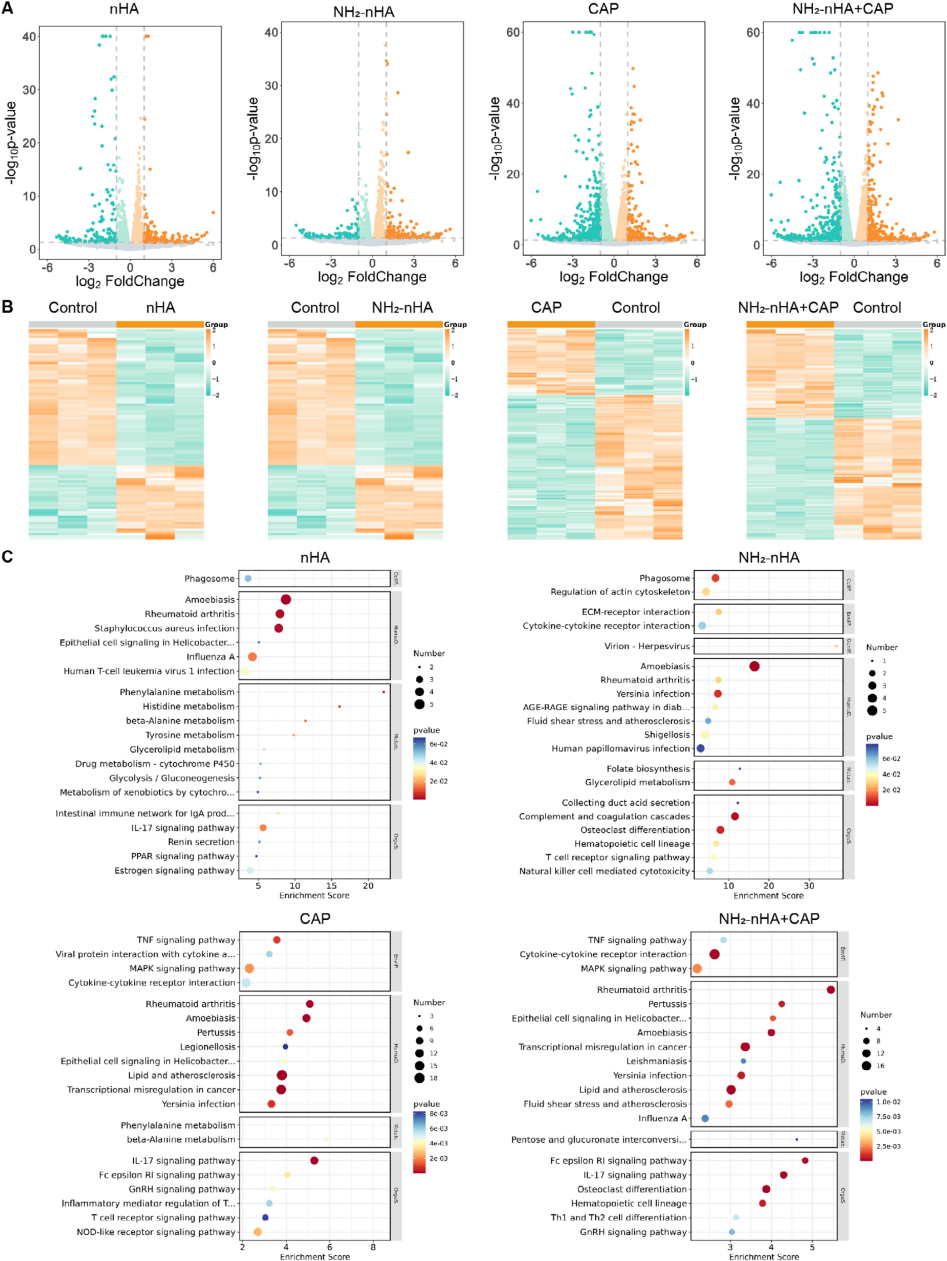
Functional enrichment analysis of CAP treatment HSC‐3 cells obtained from RNA‐seq. (A) Volcano plot of CAP, nHA and NH_2_‐nHA treatment regulated genes in HSC‐3 cells. (B) A heatmap of differentially expressed genes in CAP, nHA and NH_2_‐nHA treatment and control in HSC‐3 cells that was generated using R software, respectively. (C) The signalling pathway based on KEGG enrichment analysis of signal transduction pathway sub‐categories.

To further investigate the effect of NH_2_‐nHA and CAP on OSCC cells, we separately treated HSC‐3 cells with NH_2_‐nHA and CAP. Transcriptome sequencing was performed, followed by differential gene analysis. Heatmaps and volcano maps of the results are shown in Figure 6A,B. In HSC‐3 cells, we identified 420 differentially expressed genes in the CAP treated group, 454 in the NH_2_‐nHA+CAP treated group, 52 in the NH_2_‐nHA treated group, and 96 in the nHA treated group. KEGG enrichment analysis revealed the involvement of differentially expressed genes in various pathways, such as phagosome, TNF signalling pathway, cytokine‐cytokine receptor interaction, MAPK signalling pathway, IL‐17 signalling pathway (Figure 6C). GO enrichment analysis results were shown in Figure S12. These results of transcriptome sequencing indicated that these genes are primarily associated with extracellular matrix organisation, angiogenesis, inflammatory response and MAP kinase tyrosine phosphatase activity. To some extent, these RNA sequencing findings provide valuable insights for our subsequent experiments.

**FIGURE 7:**
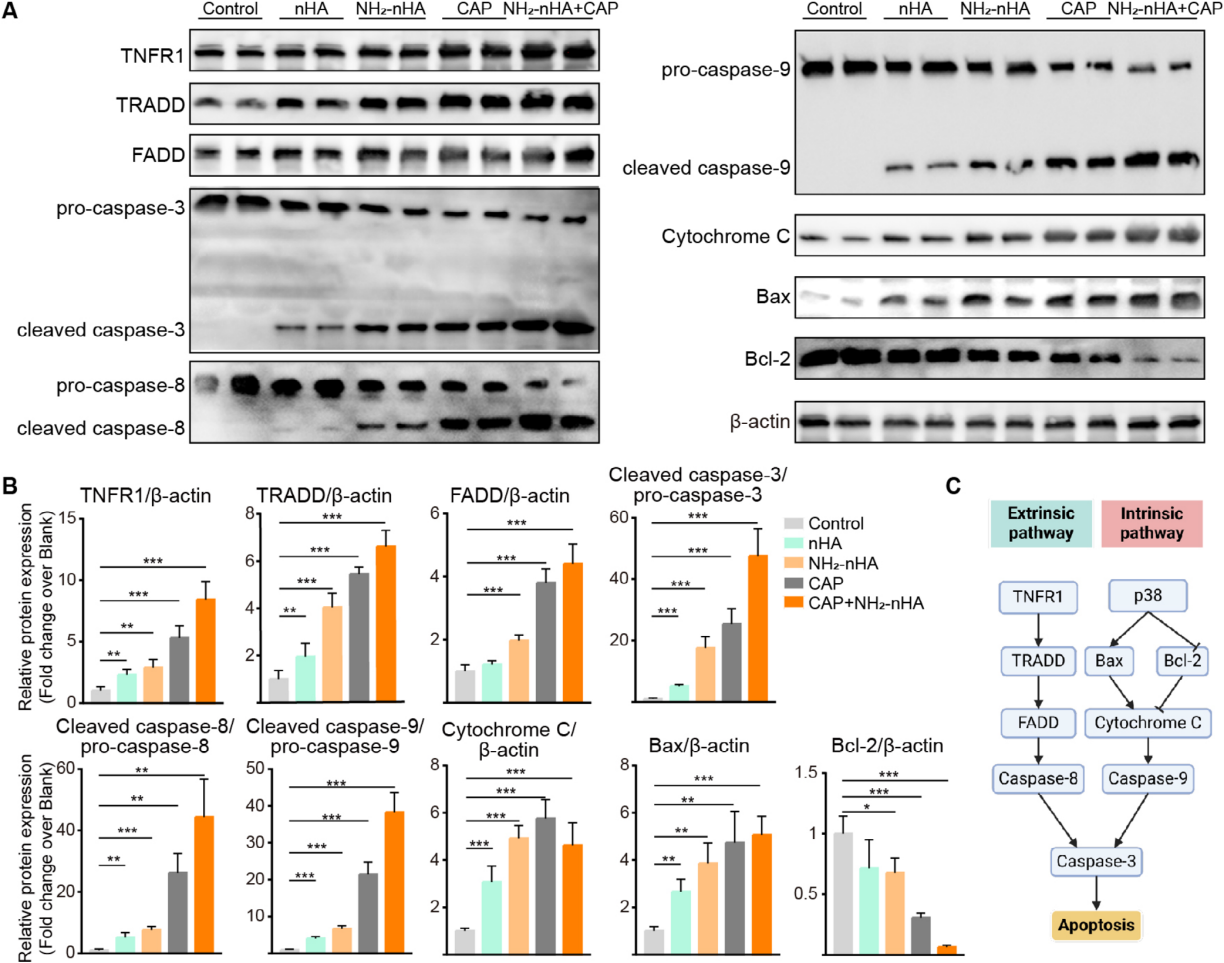
CAP and NH_2_‐nHA treatment increase the extrinsic and intrinsic pathways of apoptosis in HSC‐3 cells. (A) Protein expression of TNFR1, TRADD, FADD, cleaved caspase‐3, pro‐caspase‐3, cleaved caspase‐8, pro‐caspase‐8, cleaved caspase‐9, pro‐caspase‐9, cytochrome C, bax and bcl‐2 were analysed through Western blotting. (B) The bands were quantified using Image J software, and the results are evident from the histogram. β‐Actin was utilised as a loading control (The data are presented as mean ± SD, *n* = 3, not labelled: *p* > 0.05, *p ≤ 0.05, **p ≤ 0.01, ***p ≤ 0.001). (C) The apoptosis pathway: extrinsic pathway and intrinsic pathway.

Following the previous findings, we investigated the effects of NH_2_‐nHA and CAP on the extrinsic and intrinsic apoptotic pathway of HSC‐3 cells in Figure 7. Western blot analyses demonstrated significant increased protein expression of TNFR1, TRADD, FADD, caspase 3, caspase 8, caspase 9, cytochrome C and bax in the CAP group and NH_2_‐nHA+CAP group. Bcl‐2 showed significant decreased protein expression in the CAP group and NH_2_‐nHA+CAP group. A study by Yuan et al. highlighted that the antitumor activity and apoptosis‐inducing effects of nHA are significantly influenced by particle size. Comparing the effects of nHA particles ranging from 45 to 175 nm on liver cancer cells, they found that nHA particles sized between 20 and 80 nm effectively activated caspase‐3 and caspase‐9, reduced Bcl‐2 protein levels, and increased the levels of Bax, Bid, and the release of cytochrome C from the mitochondria into the cytoplasm. Notably, nHA particles of 45 nm demonstrated the highest efficacy in inducing apoptosis in liver cancer cells [65]. Research by Dong et al. further confirmed that the dissolution of nHA within tumour cells leads to intracellular calcium overload, thereby activating the mitochondrial apoptosis pathway and exerting tumour‐suppressive effects [38].

Apoptosis is initiated by two main pathways: the extrinsic (cell death receptor) and intrinsic (mitochondrial) pathways [66]. In the extrinsic pathway, death ligands bind to death receptors, activating the death receptor‐FADD‐caspase‐8 pathway. The intrinsic pathway is triggered by internal stress like hypoxia, DNA damage, and so forth. BCL‐2 family members regulate membrane permeability. Cytochrome C release activates caspase 3 and forms apoptosomes [67]. In the extrinsic pathway, death receptors (e.g., TRAIL, TNF receptors) on the cell surface are activated by ligands, leading to receptor oligomerization, recruitment of TRADD and FADD, and activation of caspase‐8. Caspase‐8 can cleave caspase‐3 or cleave Bid to tBid, which activates the intrinsic pathway. In the intrinsic pathway, stressors cause Bax translocation to mitochondria, along with tBid, leading to mitochondrial outer membrane permeabilisation, cytochrome C release, and activation of caspase‐9 and caspase‐3.

Therefore, we explored the potential mechanisms by which CAP promotes OSCC cell apoptosis. Through assays for cell apoptosis and cell cycle, intracellular calcium and ROS detection, mitochondrial fluorescence imaging and gene expression analysis, we found that CAP intervention leads to an increase in intracellular ROS in HSC‐3 cells, causing increased Ca^2+^ efflux from the endoplasmic reticulum. The resulting intracellular calcium overload promotes mitochondrial membrane potential loss, creating a ROS‐Ca^2+^ feedback loop that activates the caspase‐9 mitochondrial apoptosis pathway, thereby inhibiting tumour growth. To further optimise and enhance the tumour‐suppressive effects of CAP, we targeted calcium and designed a tumour microenvironment pH‐responsive NH_2_‐nHA for combined intervention with CAP. In vitro experiments showed that after NH_2_‐nHA is phagocytosed by cells and dissolved, it releases calcium ions, accelerating intracellular calcium overload and subsequently activating cell apoptosis. In this study, NH_2_‐nHA+CAP could effectively promote both death receptor (extrinsic) and mitochondrial (intrinsic) apoptosis pathways.

### 3.7 In Vivo Anti‐Tumour Study

**FIGURE 8:**
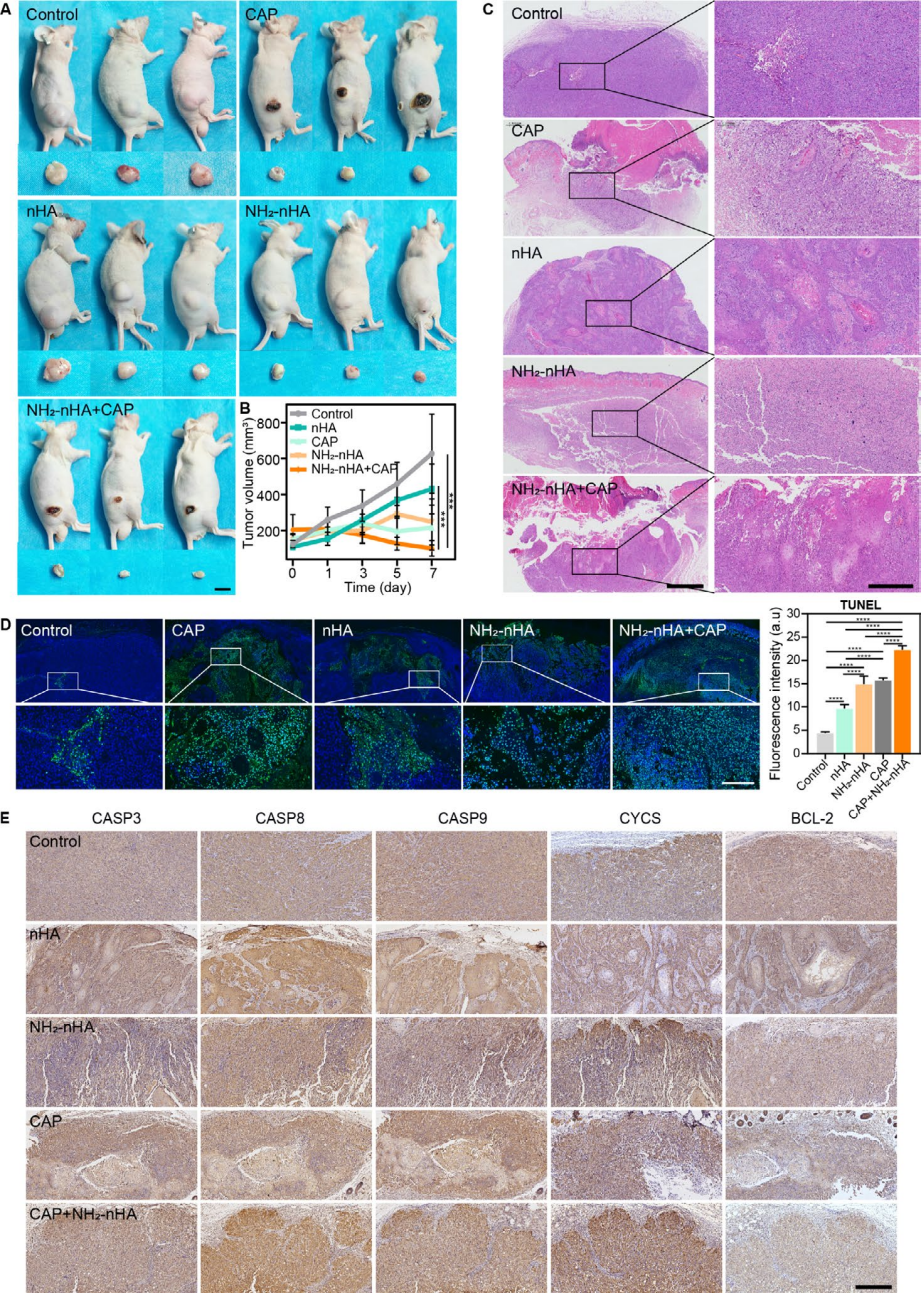
CAP, nHA and NH_2_‐nHA synergistically treatment affects tumour growth in vivo. (A) Images of mouse tumour‐bearing (scale bar 1 cm). (B) Statistical analysis of tumour volume. (C) Representative HE staining of tumours (scale bar 200 μm). (D) Representative TUNEL staining of tumours and quantification of TUNEL fluorescence intensity (scale bar 200 μm). (E) Immunohistochemical staining of apoptotic‐related proteins in tumour tissues. Statistical analysis of immunohistochemistry in Figure S17 (not labelled: *p* > 0.05, ***p ≤ 0.001, ****p ≤ 0.0001).

The HSC‐3 cells were injected into the lower back of the mice to induce tumours. Once the tumours reached an arbitrary volume of 100 mm^3^, interventions were applied to the tumours. As shown in Figure 8A, the CAP group and CAP+NH_2_‐nHA group exhibited “scab‐like” changes on the surface of the tumours, and the scab range and morphology were consistent with the range and morphology of the tumours. But “scab‐like” changes were not observed on the surrounding skin. Tumours of the CAP+NH_2_‐nHA group significantly contracted and flattened after 7 days. The statistical analysis of tumour volume in Figure 8B indicated that the average tumour volumes at 7 days are: Control group 627.28 mm^3^, nHA group 430.81 mm^3^, NH_2_‐nHA group 247.91 mm^3^, CAP group 216.21 mm^3^ and CAP+NH_2_‐nHA group 100.56 mm^3^. The tumour volume significantly decreased in the CAP, NH_2_‐nHA, CAP+NH_2_‐nHA groups and nHA also slowed tumour growth, indicating the anti‐tumour effect of CAP and NH_2_‐nHA in vivo.

HE staining of tumour tissues in each group was shown in Figure 8C. In the control group, tumour cells were densely arranged with large nuclei, and small areas of liquefied necrosis were visible in the central. In the CAP group, tumour cells were loosely arranged with condensed nuclei. In the nHA group, clusters of poorly differentiated keratinised epithelial cells were visible, surrounded by tumour cells with deeply stained and condensed nuclei. In the NH_2_‐nHA and CAP+NH_2_‐nHA groups, a large number of tumour cells with deeply stained and condensed nuclei were distributed throughout the tumour, indicating apoptosis. To further confirm the state of cell death within the tumours, TUNEL staining was used to show apoptotic cells in Figure 8D. In the control group, only a few apoptotic cells were visible in the central part of the tumour. In the CAP group, widespread and evenly distributed apoptotic cells were visible subcutaneously, with no obvious distribution of apoptotic cells in cancer nests. In the nHA group, apoptotic cells were only distributed in a small area subcutaneously. In the NH_2_‐nHA and CAP+NH_2_‐nHA groups, apoptotic cells were widely distributed within the tumour. According to the statistical analysis of TUNEL fluorescence intensity, the fluorescence intensity of each group was as follows: control 4.40, nHA 9.68, NH_2_‐nHA 14.92, CAP 15.96 and CAP+NH_2_‐nHA 22.26. Therefore, the NH_2_‐nHA group, CAP group and CAP+NH_2_‐nHA group can effectively promote apoptosis of tumour cells and inhibit tumour growth. There was no significant difference in body weight between the treatment group and the control group (Figure S13). In addition, the results of skin temperature imaging, entrails HE staining and blood cell analysis both indicated reliable biocompatibility of CAP and nHA/NH_2_‐nHA treatments (Figure S14–S16).

To determine the effect of CAP and NH_2_‐nHA synergistic intervention on apoptosis‐related pathways in vivo, immunohistochemical staining of apoptosis‐related proteins in the tumour was shown in Figure 8E. CASP3, CASP8, CASP9 and CYCS showed varying degrees of upregulation. Significant increases of CASP3, CASP8 and CYCS were observed in the NH_2_‐nHA and CAP+NH_2_‐nHA groups. Significant increases of CASP9 expression were observed in the NH_2_‐nHA, CAP and CAP+NH_2_‐nHA groups. Additionally, there no significant differences in BCL‐2 expression between the groups. These results indicated that CAP activated caspase‐9‐related endogenous mitochondrial apoptotic pathway. NH_2_‐nHA activated both the caspase‐9‐related endogenous mitochondrial apoptotic pathway and the caspase‐8‐related exogenous death receptor apoptotic pathway. Consequently, CAP and NH_2_‐nHA synergistic intervention activated both mitochondrial and death receptor apoptotic pathways to induce HSC‐3 cells apoptosis, achieving an excellent inhibitory effect on OSCC tumours in vivo.

## 4 Conclusion

In this pioneering preclinical study, we investigate the anti‐tumour effects of microwave‐induced CAP combined with pH‐responsive NH_2_‐nHA on OSCC cells, revealing novel molecular mechanisms. We demonstrated that CAP augments extracellular ROS production, whereas NH_2_‐nHA enhances intracellular Ca^2+^ concentrations, synergistically elevating intracellular ROS levels to induce oxidative stress in OSCC cells. ROS‐mediated calcium overload and calcium‐induced ROS increases create a ROS‐Ca^2+^ synergistic amplifying loop, activating the mitochondrial apoptosis pathway and leading to the prolonged apoptotic effect on OSCC. Using an in vitro model and an in vivo model, we show that CAP+NH_2_‐nHA effectively inhibits critical cancer hallmarks, including cell proliferation, cell cycle, oxidative stress, mitochondrial dysfunction, and apoptosis. Our findings highlight CAP+NH_2_‐nHA's dual action: it not only induces diverse ways of OSCC cells apoptosis but also creates a ROS‐Ca^2+^ synergistic amplifying loop. Bioinformatics analysis identified key signalling pathways affected by CAP+NH_2_‐nHA, including the TNF signalling pathway and the MAPK signalling pathway. Additionally, our results indicate a synergistic potential when CAP is combined with nanoparticles, enhancing clinical applicability. In conclusion, CAP+NH_2_‐nHA represents a promising neoadjuvant therapy for OSCC, offering a selective, accurate, portable and non‐invasive potential strategy for clinical trials in human patients.

## Author Contributions

**Wenting Qi:** writing – review and editing, writing – original draft, visualisation, validation, supervision, resources, methodology, formal analysis, data curation, conceptualization. **Hanghang Liu:** writing – review and editing. **Huaze Liu:** visualisation, validation, software, methodology, investigation, formal analysis. **Yuxuan Guo:** software, investigation, formal analysis, data curation. **Li Wu:** writing – review and editing, validation, supervision, resources, project administration, funding acquisition, conceptualization. **Chongyun Bao:** writing – review and editing, validation, supervision, resources, project administration, funding acquisition, conceptualization. **Xian Liu:** writing – review and editing, validation, supervision, resources, project administration, methodology, investigation, data curation, funding acquisition, conceptualization.

## Ethics Statement

Research Ethics Committee of West China Hospital of Stomatology, No.: WCHSIRB‐D‐2021‐630.

## Conflicts of Interest

The authors declare no conflicts of interest.

## Supporting information


Data S1.

## Data Availability

All data associated with this study are presented in the paper or the Supporting Information. The data that support the findings of this study are available from the corresponding author upon reasonable request.
