# Supplementary material for: Synergistical Induction of Apoptosis via Cold Atmospheric Plasma and Nanohydroxyapatite for Selective Inhibition of Oral Squamous Cell Carcinoma in Tumour Microenvironment
Source: Cell Prolif. 2025 Apr 29;58(10):e70041. doi: 10.1111/cpr.70041 (PMC12508692; doi:10.1111/cpr.70041)
Supplement: Supplementary file 1 — Data S1. [file CPR-58-e70041-s001.docx]

**Supplementary Information**


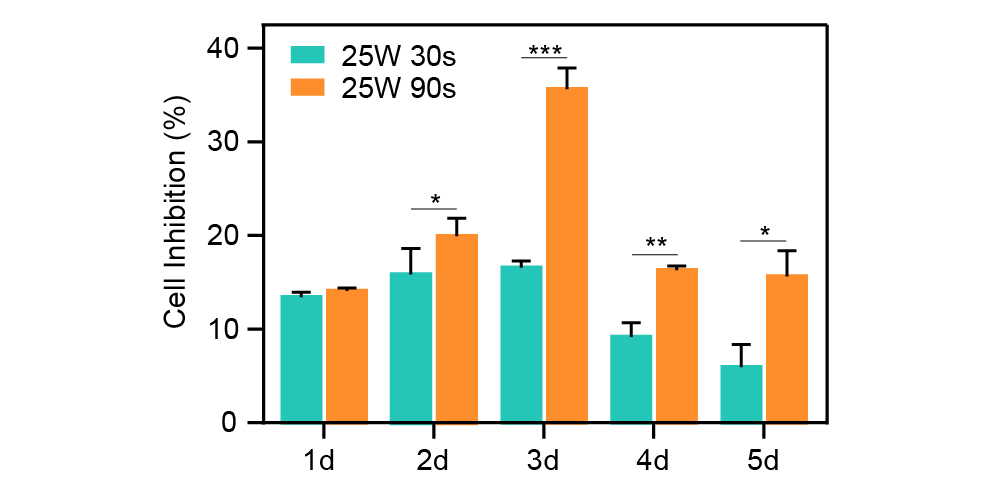


**F****igure S1** Cell inhibition rate of HSC-3 cells from 1 to 5 days under different powers of CAP treatment. (not labeled: p>0.05, *p≤0.05, **p≤0.01, ***p≤0.001, ****p≤0.0001)


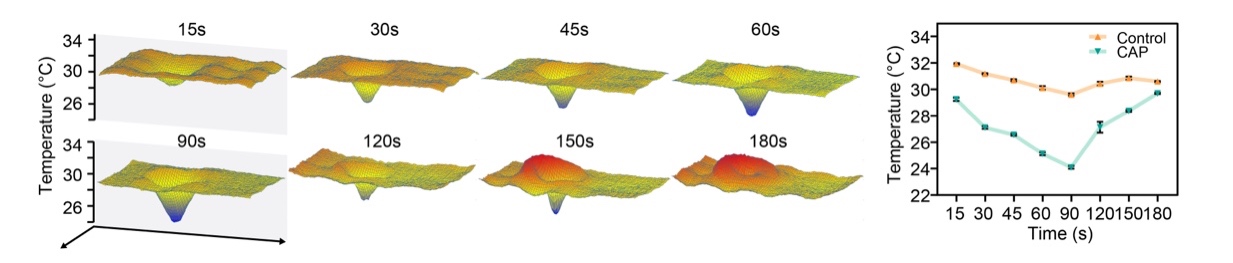


**Figure S****2** The temperature of cell culture plate changed with 35w CAP treatment 15s to 180s.


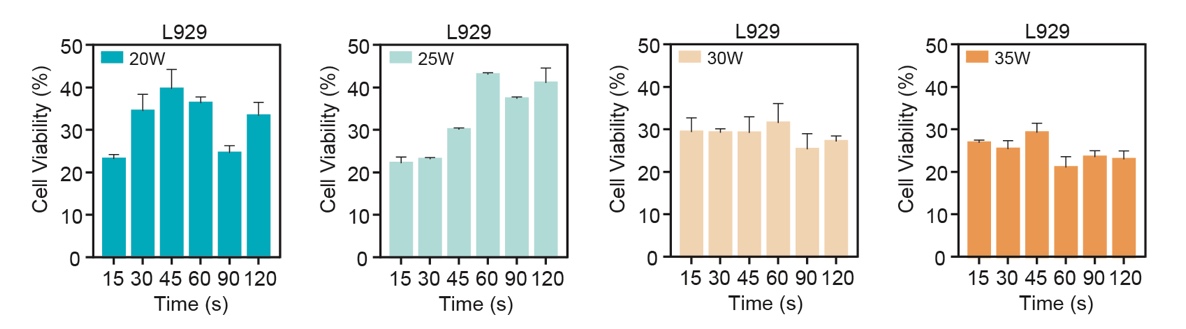


**Figure** **S3** Cell viability rate of L929 cells at different powers on day 3.


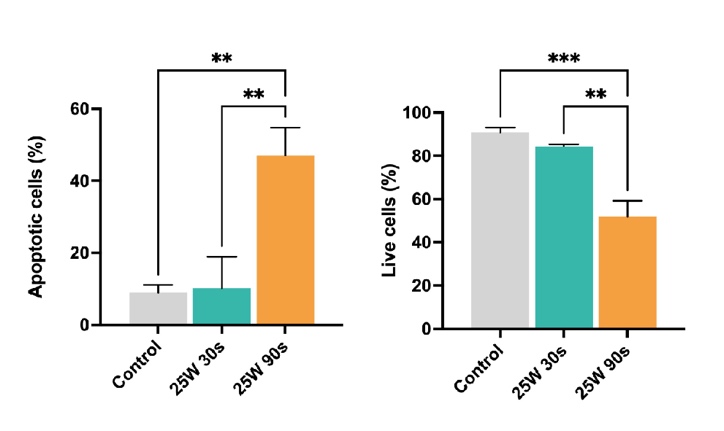


**Figure S4** Statistical analysis of the apoptosis of HSC-3 cells of Figure 2B. (not labeled: p>0.05, *p≤0.05, **p≤0.01, ***p≤0.001, ****p≤0.0001)


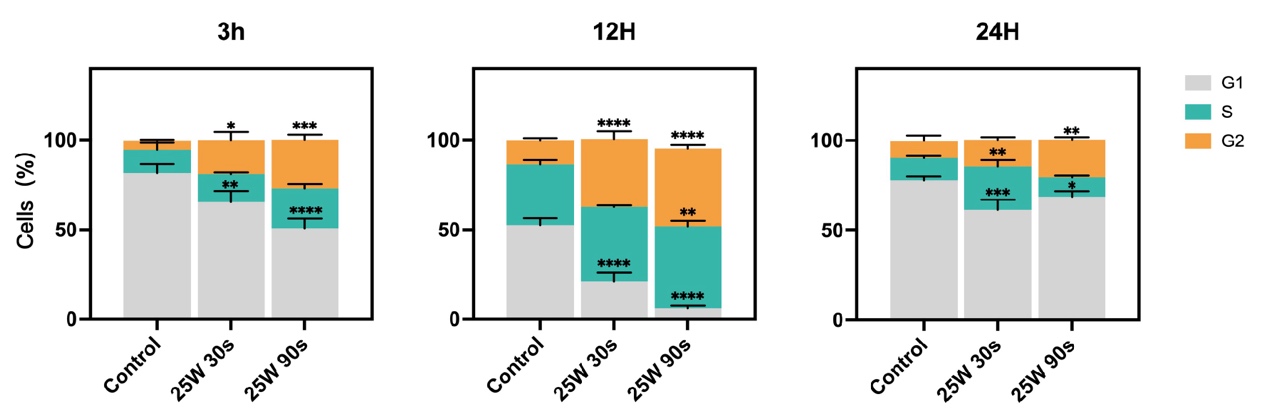


**Figure S5** Statistical analysis of the cell cycle progression of HSC-3 cells of Figure 2D. (compared with control, not labeled: p>0.05, *p≤0.05, **p≤0.01, ***p≤0.001, ****p≤0.0001)


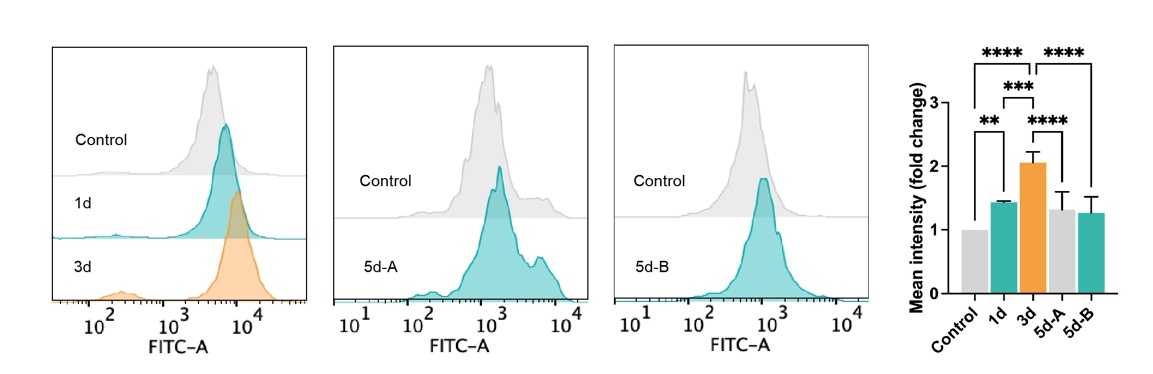


**Figure S6** Flow cytometry analysis of the ROS level of HSC-3 cells induced by CAP treatment for 1 to 5 days. 5d-A: the culture medium of HSC-3 cells was removed and exchanged into fresh culture medium on day 3 after CAP treatment. 5d-B: the culture medium of HSC-3 cells was not removed until day 5 after CAP treatment. (not labeled: p>0.05, *p≤0.05, **p≤0.01, ***p≤0.001, ****p≤0.0001)


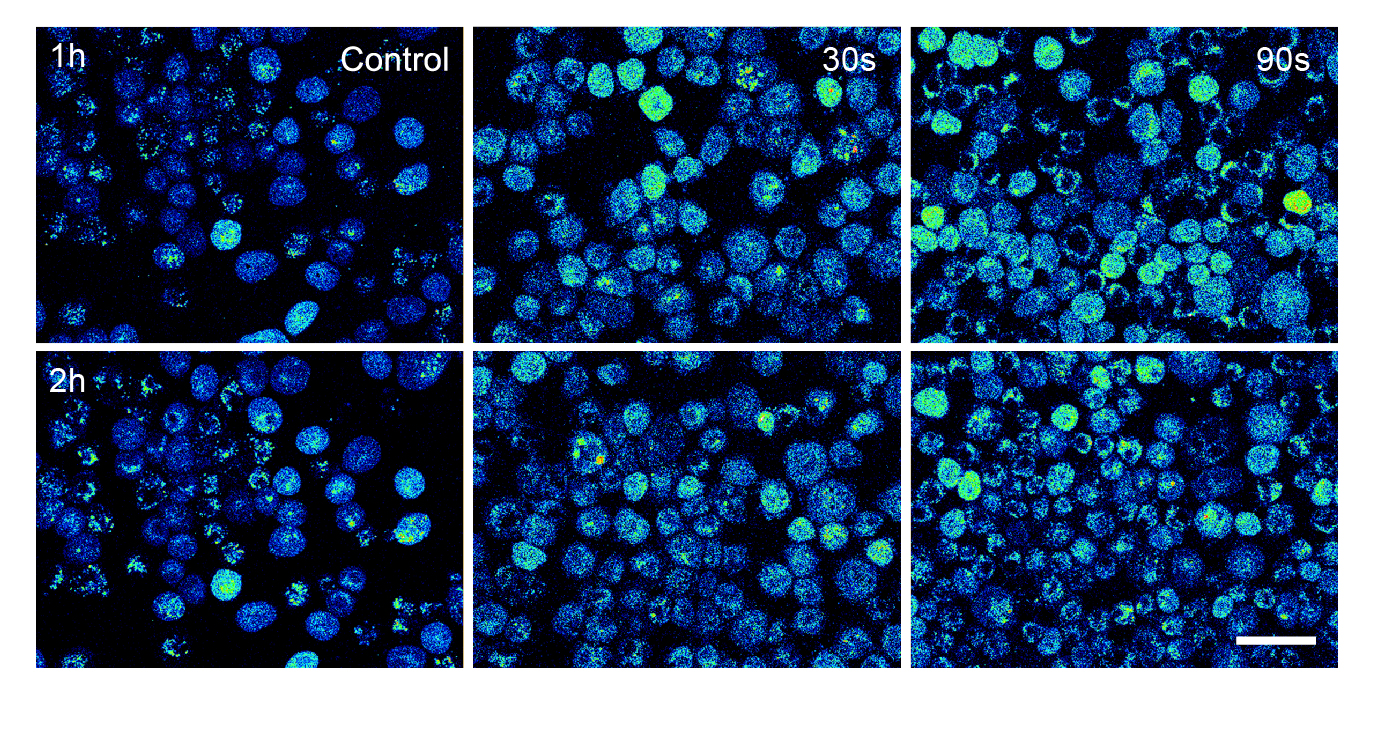


**Figure** **S7**Fluorescence images of calcium-sensing dye (Fluo-4 AM) of HSC-3 after CAP treatment for 1 h to 2 h (scale bar 50 μm).


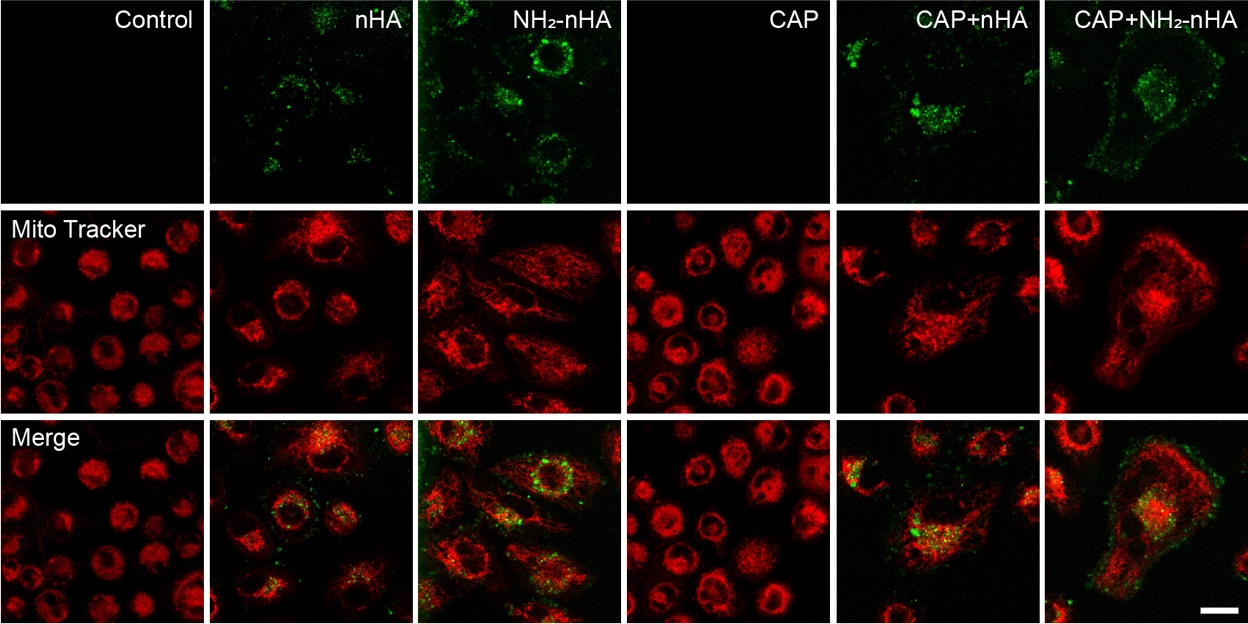


**Figure** **S8** Fluorescence images of mitochondira staining of HSC-3 cells after CAP and nHA/NH_2_-nHA treatment. Mitochondira: Mito Tracker (red). nHA, NH2-nHA(green) (scale bar 20 μm).


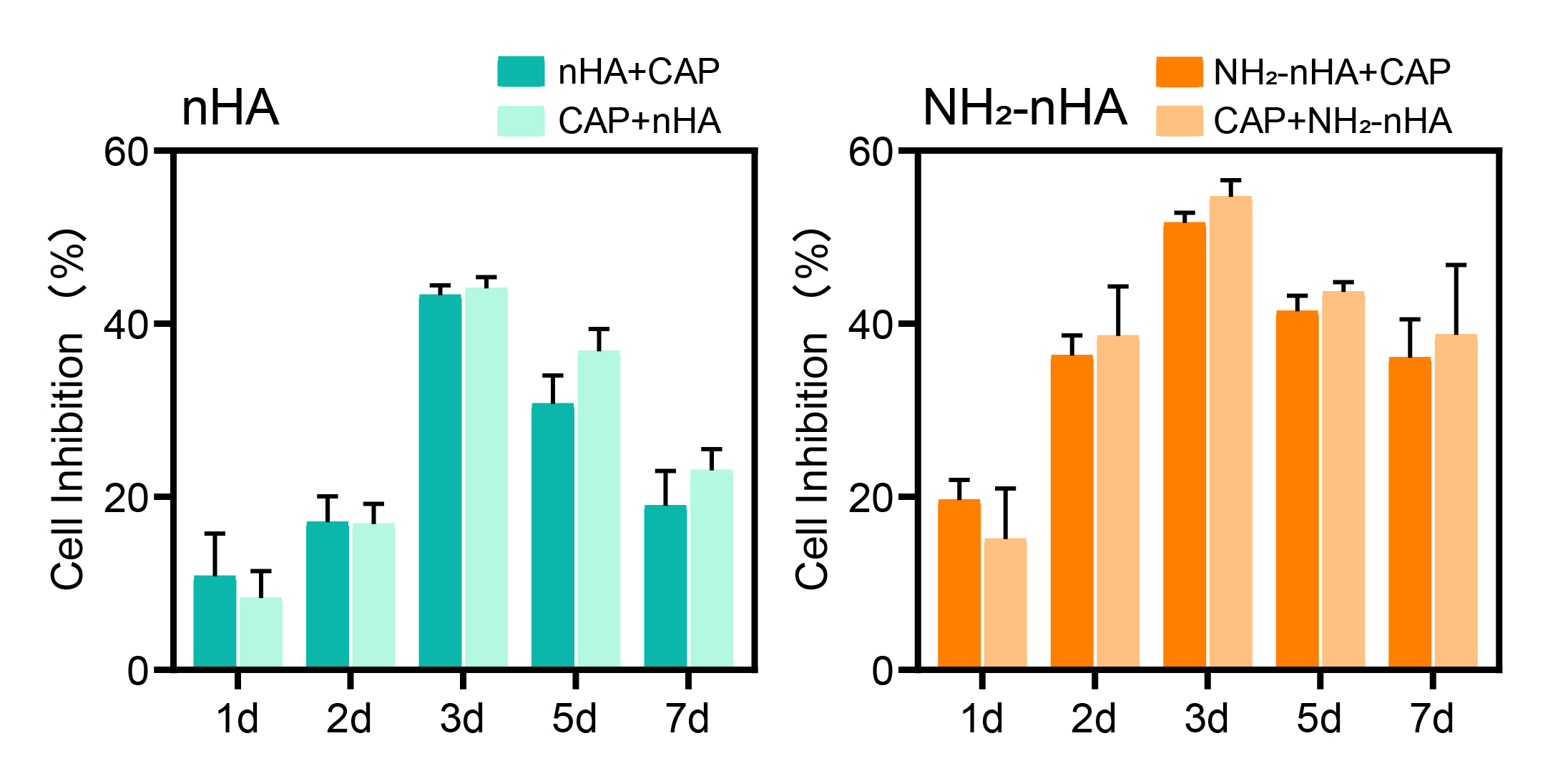
**Figure** **S9** Cell inhibition rate of HSC-3 cells from 1 to 7 days under different intervention sequences of CAP and nHA/NH_2_-nHA treatment. (not labeled: p>0.05, *p≤0.05, **p≤0.01, ***p≤0.001, ****p≤0.0001)


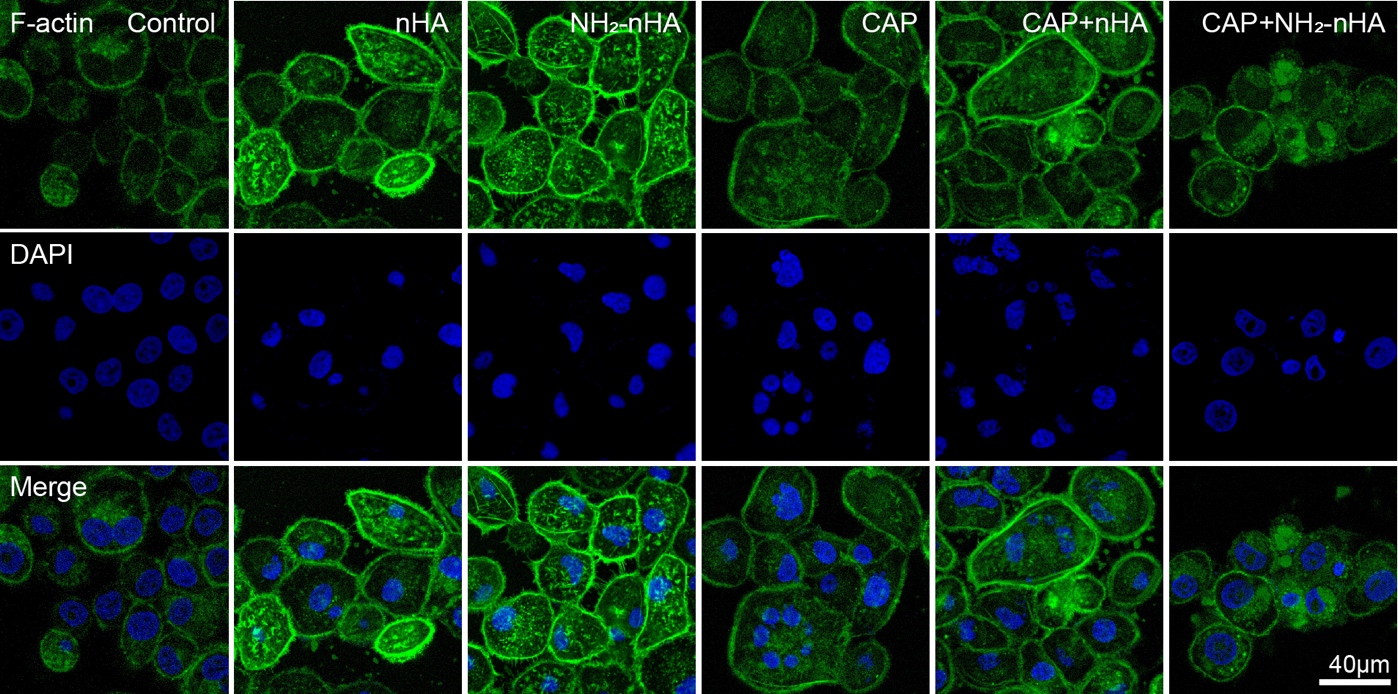


**Figure** **S10** Fluorescence images of cytoskeleton staining of HSC-3 cells after CAP treatment on 3 days. cytoskeleton: F-actin (green). nuclei: DAPI (blue) (scale bar 40 μm).


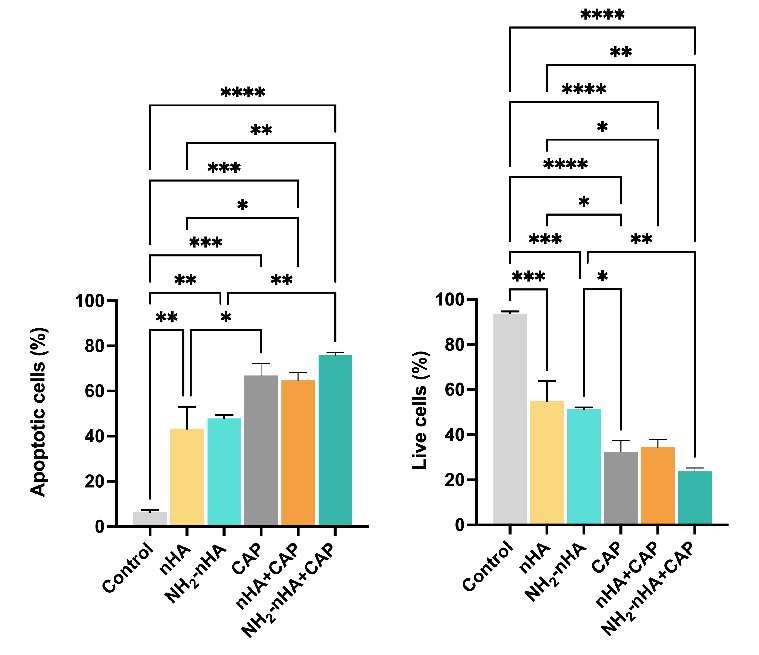


**Figure S11** Statistical analysis of the apoptosis of HSC-3 cells of Figure 5B. (not labeled: p>0.05, *p≤0.05, **p≤0.01, ***p≤0.001, ****p≤0.0001)


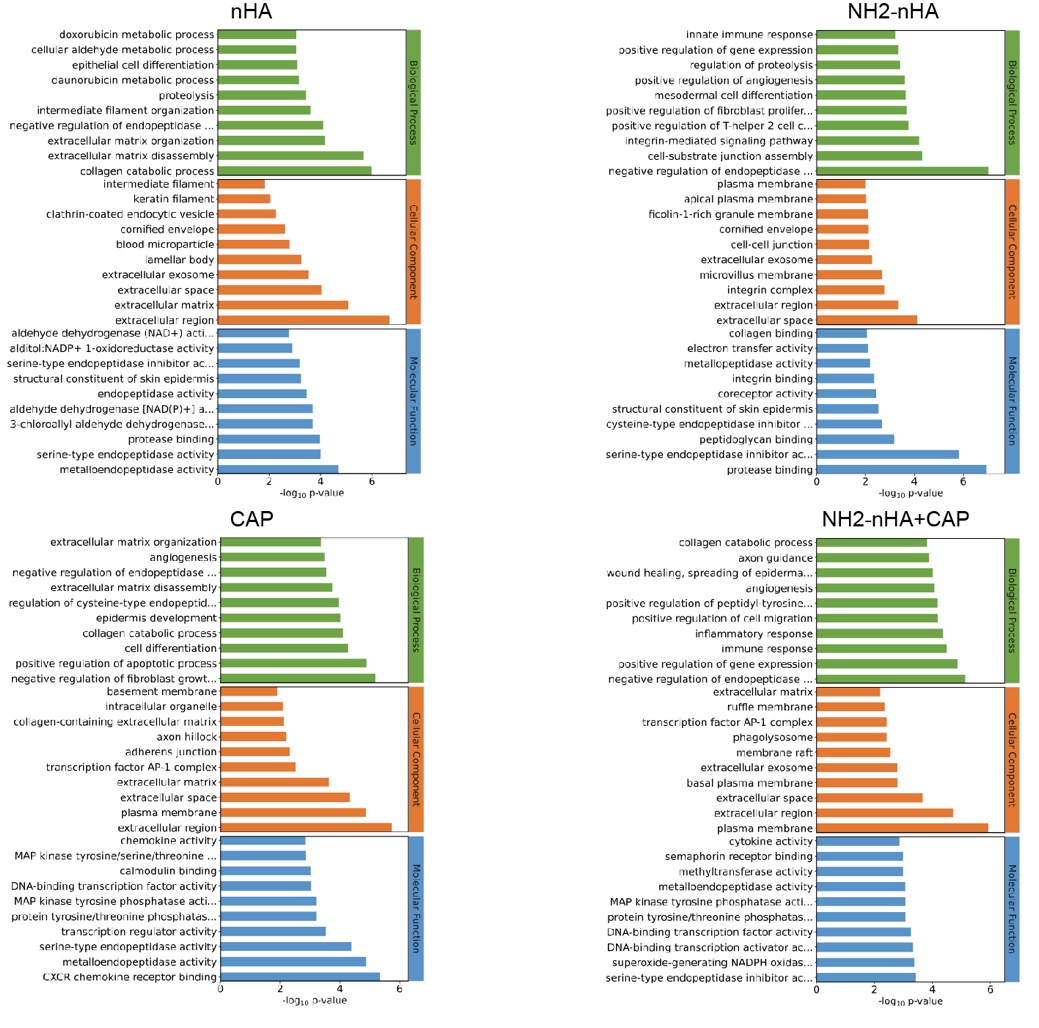


**Figure S12** The functional category based on gene ontology (GO) term enrichment.


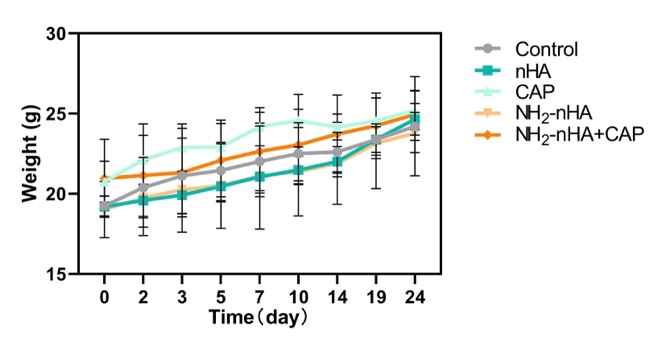


**Figure S13** Curve of body weight changes in mice. (not labeled: p>0.05, *p≤0.05, **p≤0.01, ***p≤0.001, ****p≤0.0001)


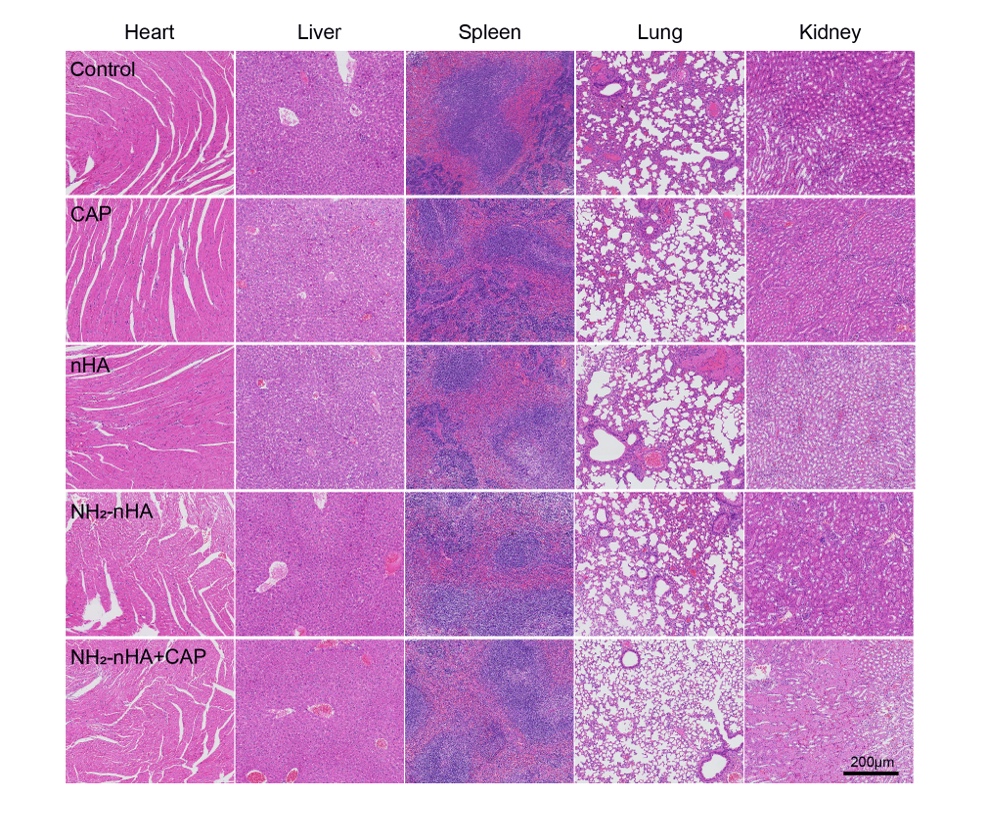


**Figure S14** Representative HE staining results of heart, liver, spleen, lung and kidney tissues of control and treated groups (scale bar 200 μm).


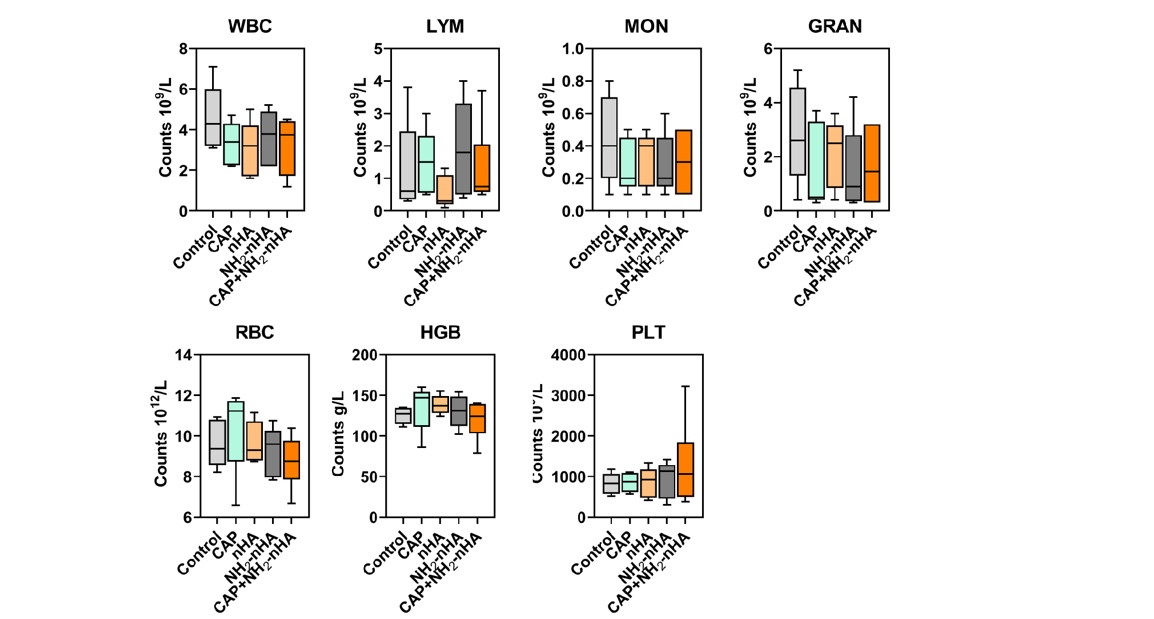
**Figure S15** The results of blood cell analysis of control and treated groups. (not labeled: p>0.05, *p≤0.05, **p≤0.01, ***p≤0.001, ****p≤0.0001)


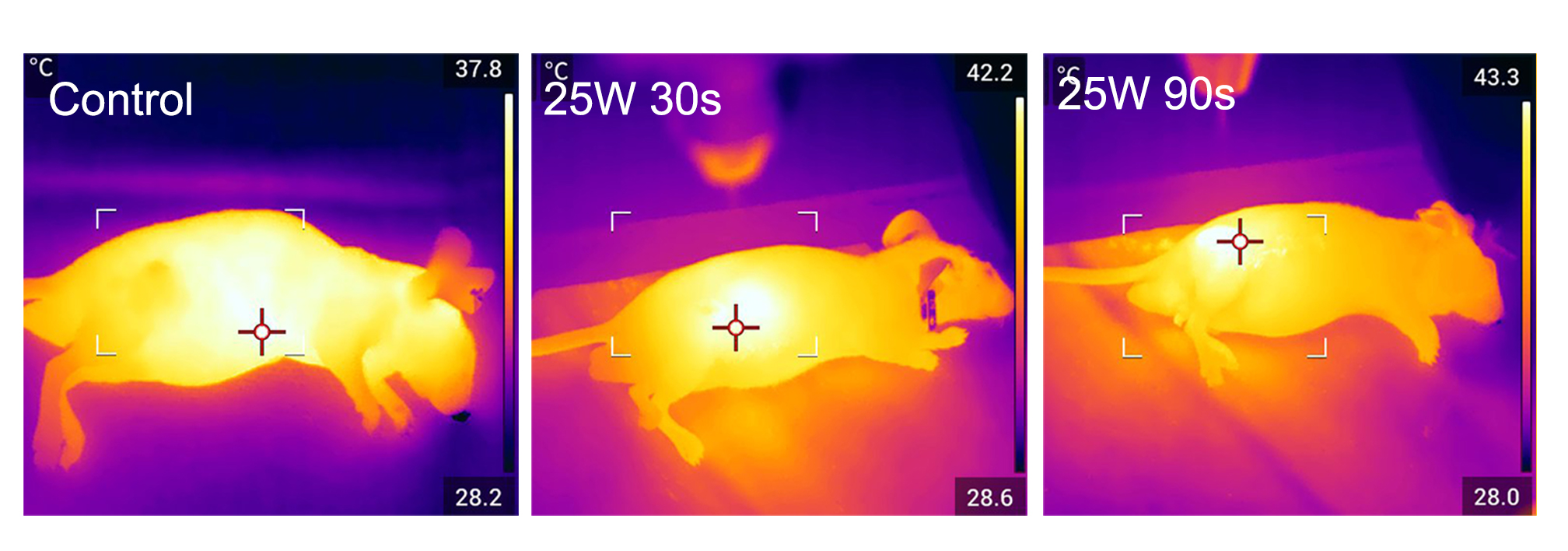


**Figure S16** Thermal imaging camera shows mouse skin temperature during the treatment of CAP.


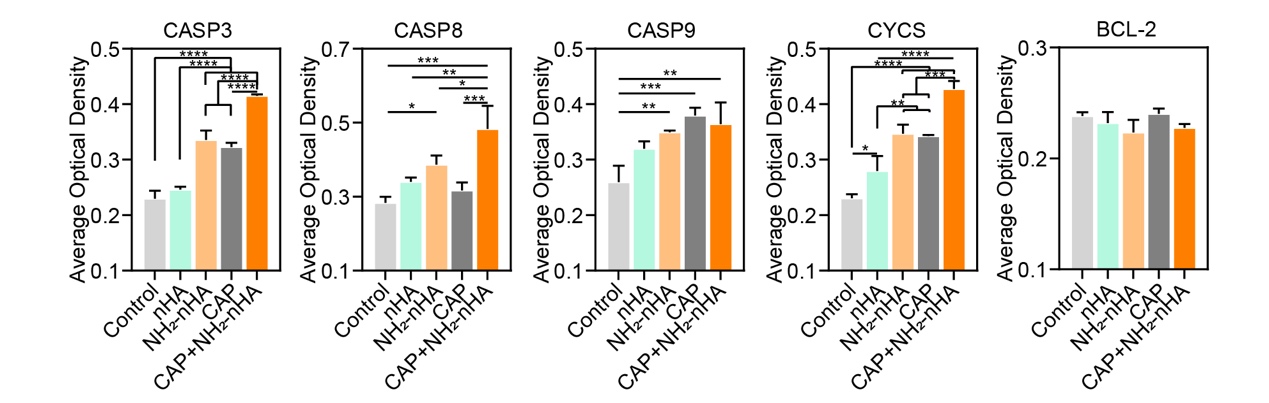
 **Figure S17** Statistical analysis of immunohistochemistry of Figure 8E. (not labeled: p>0.05, *p≤0.05, **p≤0.01, ***p≤0.001, ****p≤0.0001).

Experimental

Materials

Cell Counting Kit-8 (CCK-8, C0037), Hoechst 33342 (C1025), Actin-Tracker Green-488 (C2201S), Fluo-4 AM (S1060), 2’,7’-dichlorofluorescein diacetate (DCFH-DA, S0033), DiD (C1039) and a TUNEL kit (C1089) were purchased from Beyotime Biotechnology Co., Ltd., Shanghai, China. An Annexin V-FITC Apoptosis Detection Kit (CA1020), a TUNEL Apoptosis Assay Kit (T2190), MitoTracker Red CMXRos (M9940) and a Mitochondrial Membrane Potential Kit (JC-1, M8650) were purchased from Solarbio Technology Co., Ltd., Beijing, China. Polyclonal antibody of TNFR1 (21574-1-AP), TRADD (15468-1-AP), FADD (14906-1-AP), caspase-8 (13423-1-AP), caspase-9 (10380-1-AP), caspase-3 (19677-1-AP), caspase-8 (13423-1-AP), caspase-9 (10380-1-AP), Bcl-2 (12789-1-AP), Bax (50599-2-Ig) and cytochrome c (10993-1-AP) were purchased from Proteintech Co., Ltd., Wuhan, China. Calcium dinitrate tetrahydrate (Ca[NO_3_]_2_·4H_2_O), ammonium monohydric phosphate ([NH_4_]_2_HPO_4_), ammonium hydroxide (NH_3_·H_2_O), and (3-Aminopropyl)triethoxysilane (APTES) were obtained from Sinopharm Chemical Reagent Co., Ltd, Shanghai, China.

Preparation and characterizations of CAP

The plasma jet device employed in the experiments is designed based on a coaxial structure. A solid-state source (PA2425-250, the 13th Institute of China Electronics Technology Group Corporation, Shijiazhuang, China) was applied to provide microwave power at a frequency of 2450 MHz to trigger Ar gas discharge at atmospheric pressure. The microwave input power of the plasma device was adjusted from 25 W to 45 W. Ar gas (7 L/min) was supplied to the plasma device during the treatments, and the purity of the gas was greater than 99.99%. The distance between the plasma jet nozzle and the media in the 6-well plate was adjusted to 3 cm. A fiber optic spectral analyzer (Avantes AVS-RACKMOUNTUSB2, Netherlands) was used to measure the optical emission spectra of the CAP. An optical fiber is located at a distance of 10 mm from the nozzle of the plasma jet.

Preparation and characterizations of nHA

The preparation process of nHA and NH_2_-nHA were schematized in Figure 4A. The morphologies and internal structures were investigated by using a field-emission scanning electron microscopy (FE-SEM) (Hitachi S-4800, Japan) and a field-emission transmission electron microscopy (FE-TEM) (JEM-2100, Japan). X-ray diffraction (XRD, Rigaku D/max2550 VB/PC, Japan) was employed to analyze the phase compositions and crystallinities of the prepared nanoparticles under 40 kV/100 mA and 3500 CPS for Cu target. The chemical structure was analyzed by Fourier transform infrared spectroscopy (FTIR, Nicolet Magma-550 series II, Midac, USA). The zeta potentials at different pH were monitored and recorded by Zetasizer Nano ZS (Malvern Instruments Ltd, UK).

Cell culture

The HSC-3 cell line for human oral squamous cell carcinoma was acquired from the West China Hospital of Stomatology, Sichuan University's State Key Laboratory of Oral Diseases and National Center for Stomatology. HSC-3 cells were grown in Dulbecco's modified Eagle's medium (DMEM, Gibco, Life Technologies, Carlsbad, CA, USA) supplemented with 10% fetal bovine serum at 37°C in a humid environment with 5% CO_2_.

Cell viability assay

The CCK-8 assay was utilized to assess the viability of HSC-3 and L929 cells in vitro. A total of 3×10^3^ cells per well were cultured in 96-well plates for assembly. After an overnight incubation period and CAP treatment lasting one to five days, 100 mL of media supplemented with 10 mL of the CCK-8 reagent was added to each well, and the plates were incubated at 37°C for two hours. To measure the absorbance (A) at 450 nm, a microplate reader (Multiskan FC, Thermo Fisher Scientific, Waltham, MA, USA) was used. In each group, three parallel wells were used, and the mean value was calculated.

Cell apoptosis assay

Propidium iodide (PI) is utilized for detecting necrosis, while FITC-annexin V is used for detecting apoptosis in cells after CAP treatment. After CAP treatment, the HSC-3 cells were incubated for 24 hours in a 5% CO_2_ incubator at 37°C. The cells were washed with PBS and then harvested using trypsin. The resuspended cells were then added to binding buffer at a cell density of 1×106 per mL. Apoptotic cells were stained with 5 μL of Annexin V-FITC and then incubated for 10 min. Necrotic cells were then stained with 5 μL of PI and incubated for an additional 5 min. Using a FACScan flow cytometer (BD Biosciences, Franklin Lakes, NJ, USA) with an excitation laser line at 488 nm for FITC-annexin and a 575 ± 15 nm bandpass filter for PI, we examined the behavior of apoptosis/necrosis in cells. All apoptosis/necrosis dye staining procedures were performed in the dark.

Measurement of cell morphology

The HSC-3 cells were fixed with paraformaldehyde for 15 min and stained with Actin-Tracker Green or DiD for 30 min. Subsequently, the nuclei were stained with DAPI. After the cells were washed with PBS, fluorescence was detected using confocal laser scanning microscopy (Zeiss LSM980, Jena, Germany). ImageJ software was used to analyze the fluorescence intensity.

Measurement of mitochondrial membrane potential

The sensitive florescence probe JC-1 is generally used to detect mitochondrial membrane potential (MMP). The cells were rinsed once with PBS and then incubated with 500 μl of 10 μg/mL JC-1 working solution in the dark for 30 min. The cells were subsequently washed twice with PBS, and the fluorescence was examined using laser confocal microscopy (ZEISS LSM980, Jena, Germany). ImageJ software was used to analyze the fluorescence intensity.

Measurement of intracellular ROS and the mitochondria network

Intracellular ROS were detected using the oxidation-sensitive fluorescent probe DCFH-DA. Mitochondria were detected with Mito-Tracker Red CMXRos. After treatment with or without CAP, the cells were rinsed twice in PBS. Next, the cells were incubated with 10 µmol/L DCFH-DA according to the manufacturer’s instructions. DCFH-DA is intracellularly deacetylated by nonspecific esterase and subsequently oxidized by ROS to the fluorescent compound 2,7-dichlorofluorescein (DCF). Mito-Tracker Red CMXRos working solution (100 nM) was added to each well and then incubated in the dark at 37°C for 10 min. Subsequently, the cells were stained with Hoechst 33342 for 10 min. After washing with PBS, fluorescence was detected by confocal laser scanning microscopy (Zeiss LSM980, Jena, Germany). ImageJ software was used to analyze the fluorescence intensity.

Measurements of intracellular calcium ion.

After CAP treatment, Fluo-4 AM was added to the HSC-3 cells in a 24-well plate. The cells were then cultured in an incubator at 37°C for 15 min, washed twice with PBS and filtered. The fluorescence intensity at 525 nm was observed using a plate reader (Micro plate Reader; Bio-Rad), and the OD value was calculated. The experiment was independently conducted 3 times. In addition, cells in glass bottom cell culture dishes were stained with Fluo-4 AM. Then, the fluorescence was detected by confocal laser scanning microscopy (ZEISS LSM980, Jena, Germany).

RNA-sequencing and bioinformatic analysis

To examine the mRNA expression profiles of CAP and nHA treated HSC-3 cells, RNA sequencing (RNA-seq) was performed on an llumina Novaseq 6000 platform and 150 bp paired-end reads were generated. Differential expression analysis was performed using the DESeq2. Q value < 0.05 and foldchange > 2 or foldchange < 0.5 was set as the threshold for significantly differential expression gene (DEGs). Hierarchical cluster analysis of DEGs was performed using R (v 3.2.0) to demonstrate the expression pattern of genes in different groups and samples. The radar map of top 30 genes was drawn to show the expression of up-regulated or down-regulated DEGs using R packet ggradar. Based on the hypergeometric distribution, GO, KEGG pathway, Reactome and Wiki Pathways enrichment analysis of DEGs were performed to screen the significant enriched term using R (v 3.2.0), respectively. R (v 3.2.0) was used to draw the column diagram, the chord diagram and bubble diagram of the significant enrichment term.

Western blot assay

For the Western blot assay, HSC-3 cells were seeded in 6-well plates at a density of 2 × 10^5^ cells/well and incubated for 24 h. Subsequently, CAP, nHA and NH_2_-nHA treatment was performed and the cells were allowed to regenerate for 24 h. The HSC-3 cells were collected and washed with ice-cold PBS solution, then lysed in RIPA cell lysis buffer (Solaibao, Beijing, China) containing complete™ Protease Inhibitor Cocktail. Protein concentrations in the supernatant were quantified using the bicinchoninic acid (BCA) Assay (Solaibao, Beijing, China). The protein extracts were separated by 10–15 % SDS-PAGE, and the gels were transferred to PVDF membranes (Immobilon-P, CA, USA). The membranes were blocked in 5 % skimmed milk, incubated with the primary antibody overnight at 4 ◦C, washed three times in TBST, and then incubated with the secondary antibody at room temperature for 1 h. The signals were developed using an enhanced chemiluminescence (ECL) kit (Immobilon-P, CA, USA), and the bands were visualized using the ChemiDoc™ Touch Imaging System (Bio-Rad). Relative quantification was performed using β-actin as an internal reference.

Tumor xenograft model

Sixty 4-week-old male BALB/c athymic nude mice were obtained from GemPharmatech (Nanjing, China). The mice were kept on a regular 12-hour light/12-hour dark cycle, and autoclaved water and food were provided ad libitum. HSC-3 cells (1×10^6^/100 µL/mouse) were subcutaneously injected into the lower back of the mice. Animals with tumors were randomly assigned to a control or CAP treatment group (5 mice per group). The nHA or NH_2_-nHA treatment began when xenografts reach a volume of approximately 100 mm^3^. CAP treatment was administered on the 0 d, 3 d, and 6 d. Tumor growth was monitored and counted when the mice started to exhibit tumors. At the end of the study (1 weeks after treatment), the animals were sacrificed. The tumors were removed from each mouse, and the weights were measured. All animal studies were conducted in accordance with institutional guidelines and approved by the Institutional Review Board (IRB) of West China Hospital of Stomatology, Sichuan University (Chengdu, China).

Tissue pathology assessment

To evaluate tissue pathology, the tumor, heart, liver, spleen, lung, and kidney tissues were procured and subjected to fixation in 4 % paraformaldehyde for 24 h, followed by embedding in paraffin. According to the standard protocol, paraffin sections (4 μm) were dewaxed with xylene and rehydrated in ethanol. Afterward, the specimens were stained with Hematoxylin and eosin (H&E), followed by observation by bright-field microscopy. For the immunohistochemical staining, sections were stained with antibodies overnight, then incubated with secondary antibody according to the manufacturer's instructions. Antibodies included rabbit anti-human caspase-3, caspase-8, caspase-9, Bcl-2, and cytochrome c. Hematoxylin was used to stain the nucleus. The TUNEL assay was performed using a TUNEL kit according to the manufacturer’s instructions. Quantification of monochrome images of the whole tumor was performed with ImageJ software by measuring the area of positive staining.

Statistical analysis

Each expirments were performed in triplicate at least. All statistical calculations were performed using the GraphPad Prism 8 software (GraphPad Software Inc., United States). The data were presented as mean ± SD. Comparisons between two groups were made by using the unpaired Student's t-test. Comparisons between multiple groups were performed using a one-way or two-way ANOVA test. Statistical significance was determined by p < 0.05.
